# Supplementary material for: Light-programmable mechanical computing via polyaniline composite film
Source: Nat Commun. 2026 Mar 16;17:4011. doi: 10.1038/s41467-026-70425-z (PMC13136495; doi:10.1038/s41467-026-70425-z)
Supplement: Supplementary file 1 — Supplementary Information [file 41467_2026_70425_MOESM1_ESM.pdf]

Supplementary Information for

Light-Programmable Mechanical Computing via Polyaniline Composite Film

Xiunan Yan[1#], Yixiang Li[1#], Yichen Zhao[1#], Chen Pan[2✉], Shengnan Yan[1], Dong Yang[2], Gong-Jie Ruan[1], Hang Zhao[1], Fanqing Chen[1], Xing-Jian Yangdong[1], Pengfei Wang[1], Wentao Yu[2], YuekunYang[1, 3], Cong Wang[1], Bin Cheng[2], Shi-Jun Liang[1, 4, 5✉], Feng Miao[1, 5✉]

[1]Institute of Brain-Inspired Intelligence, National Laboratory of Solid State Microstructures, School of Physics, Collaborative Innovation Center of Advanced Microstructures, Nanjing University, Jiangsu Physical Science Research Center, Nanjing 210093, China.

[2] Institute of Interdisciplinary Physical Sciences, School of Physics, Nanjing University of Science and Technology, Nanjing 210094, China.

[3] School of Intelligence Science and Technology, Nanjing University, Suzhou 215163, China.

[4] Chemistry and Biomedicine Innovation Center (ChemBIC), Nanjing University, Nanjing 210093, China.

[5] Institute of Brain-Machine Interface, Nanjing University, Nanjing 210093, China.

[#]These authors contributed equally to this work.

[✉]Corresponding authors, e-mail: chenpan@njust.edu.cn, sjliang@nju.edu.cn, miao@nju.edu.cn

Supplementary Note 1. Light-responsive reversible mechanical deformation mechanism of PCF

Light-responsive deformation mechanism

The PCF's reversible deformation stems from synergistic interactions among its functional layers. The PANi-PNIPAm layer integrates photothermal PANi (exhibiting strong absorption at 645 nm with high photothermal conversion efficiency)[1] and thermoresponsive PNIPAm (with a lower critical solution temperature LCST ≈ 32°C)[2]. PDMS has a positive thermal expansion coefficient and expands at high temperatures[3, 4]. Upon illumination, PANi converts light to heat, triggering PNIPAm contraction when temperature exceeds LCST, while simultaneously inducing PDMS expansion through Ag NWs-mediated heat transfer. This differential strain generates bending deformation.

Humidity-responsive recovery mechanism

Upon moisture exposure, PNIPAm rapidly hydrates through hydrogen bonding, causing PANi-PNIPAm layer expansion that restores the PCF to its original configuration. The system demonstrates complete reversibility through alternating photothermal and hygroscopic activation.

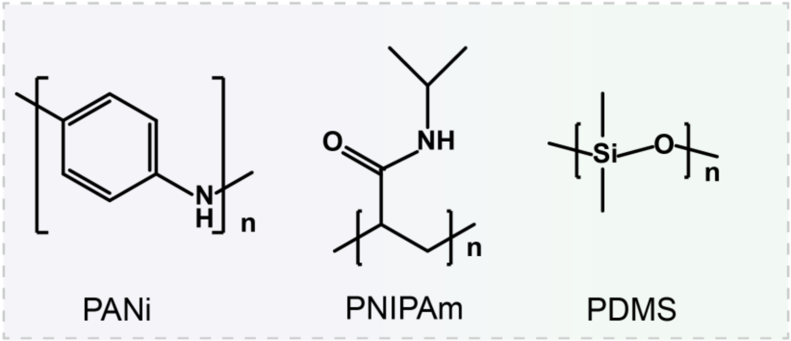

Supplementary Fig. 1. Molecular structures of the polyaniline composite film. The composite films consist of three key components: polyaniline (PANi), poly(N-isopropylacrylamide) (PNIPAm), and polydimethylsiloxane (PDMS), which collectively enable their functionality in sensing and actuation.

Supplementary Table 1. Quantitative justification for the material selection in the PDMS/Ag NWs/PANi-PNIPAm hybrid system.

Material	Functionality	Properties	References
PANi	Light-responsive / photothermal conversion	650 nm; Photothermal conversion efficiency: 49.6%	Ref[1]
PNIPAm	Thermal contraction (phase-change)<br>Humidity-Induced Swelling	Phase-change (LCST = 32 ℃)<br>Volume shrinkage: 7%-10%	Ref[2, 5]
Ag NWs	Conductive layer and heat-conductive layer	Electrical conductivity: 6.25×10^7 S/m; Thermal conductivity: 429 W/(m·K)	Ref[6, 7]
PDMS	Thermal expansion	Thermal expansion coefficient: 2.6-9.07 × 10^{-4} K^{-1}	Ref[3, 4]

This table highlights the complementary roles of each component in ensuring efficient photothermal-to-mechanical transduction, environmental responsiveness, and device stability. Importantly, the integration of these materials provides synergistic advantages that conventional single-material systems cannot achieve, thereby enabling a robust platform for light-programmable mechanical computing.

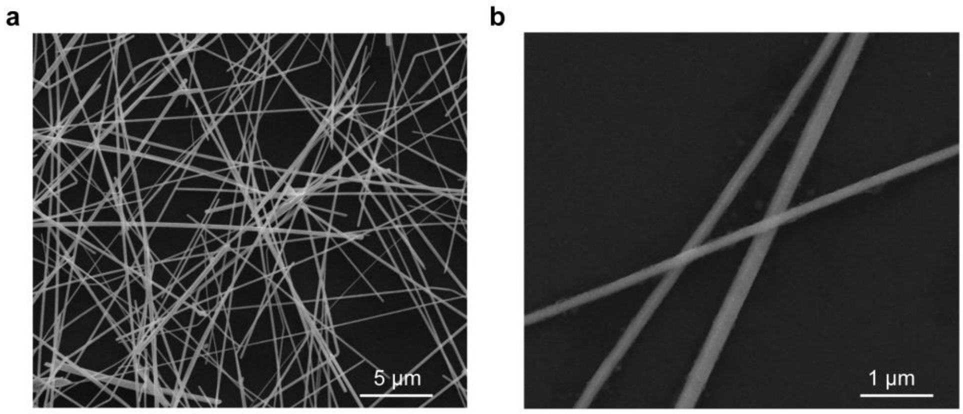

Supplementary Fig. 2. SEM images of silver nanowires (Ag NWs). a Scale bar: 5 μm. **b** Scale bar: 1 μm.

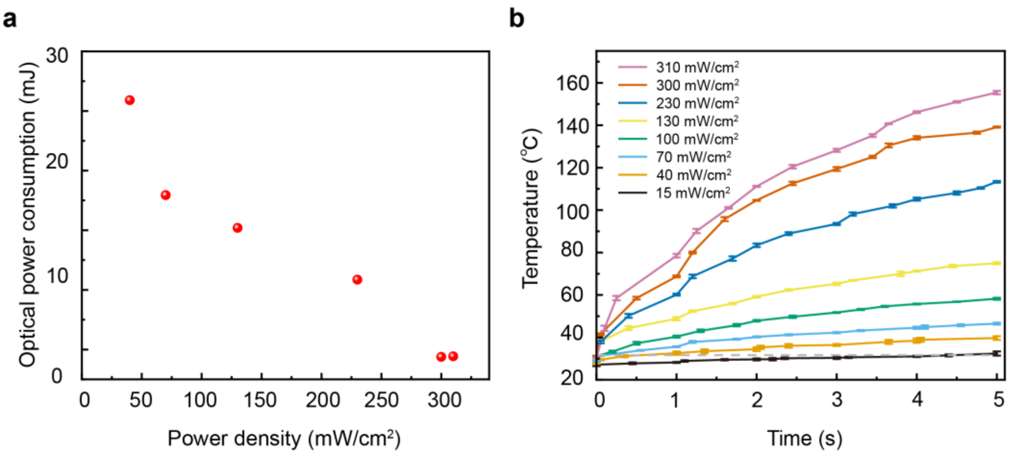

Supplementary Fig. 3. Power consumption and thermal response of the polyaniline composite film (PCF). a Power consumption per switching event under different light intensities. **b** Thermal response of the PCF (PDMS/Ag NWs/PANi–PNIPAm) under different light power intensities within 5 s of illumination. Data are represented as mean ± standard deviation (SD; n = 15). Error bars represent the SD.

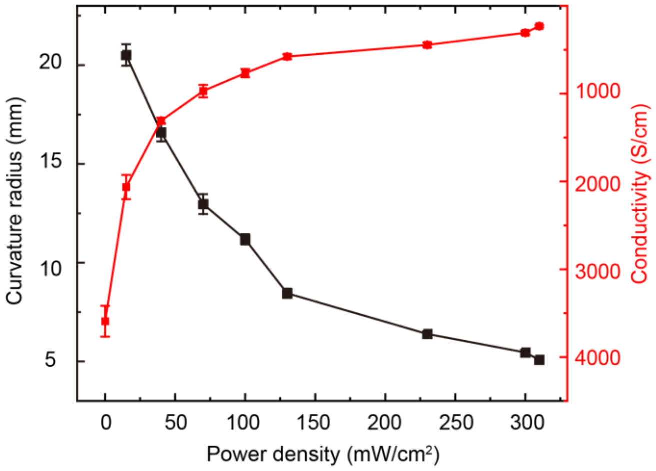

Supplementary Fig. 4. Curvature radius and conductivity changes of the polyaniline composite film (PCF) after 5 s illumination at different light intensities. The black curve represents the response time, while the red curve represents the recovery time. Data are represented as mean ± standard deviation (SD; n = 15). Error bars represent the SD.

a

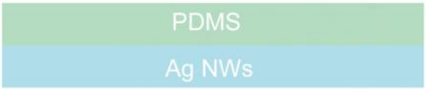

b

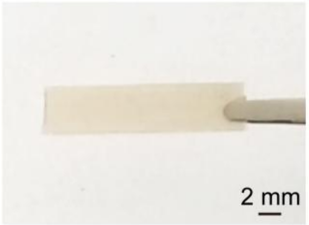

Supplementary Fig. 5. Polydimethylsiloxane/silver nanowires (PDMS/Ag NWs) composite film. **a** Schematic illustration of the PDMS/Ag NWs structure, with Ag NWs in sky blue and PDMS in light green. **b** The corresponding optical image of the PDMS/Ag NWs. Scale bar: 2 mm.

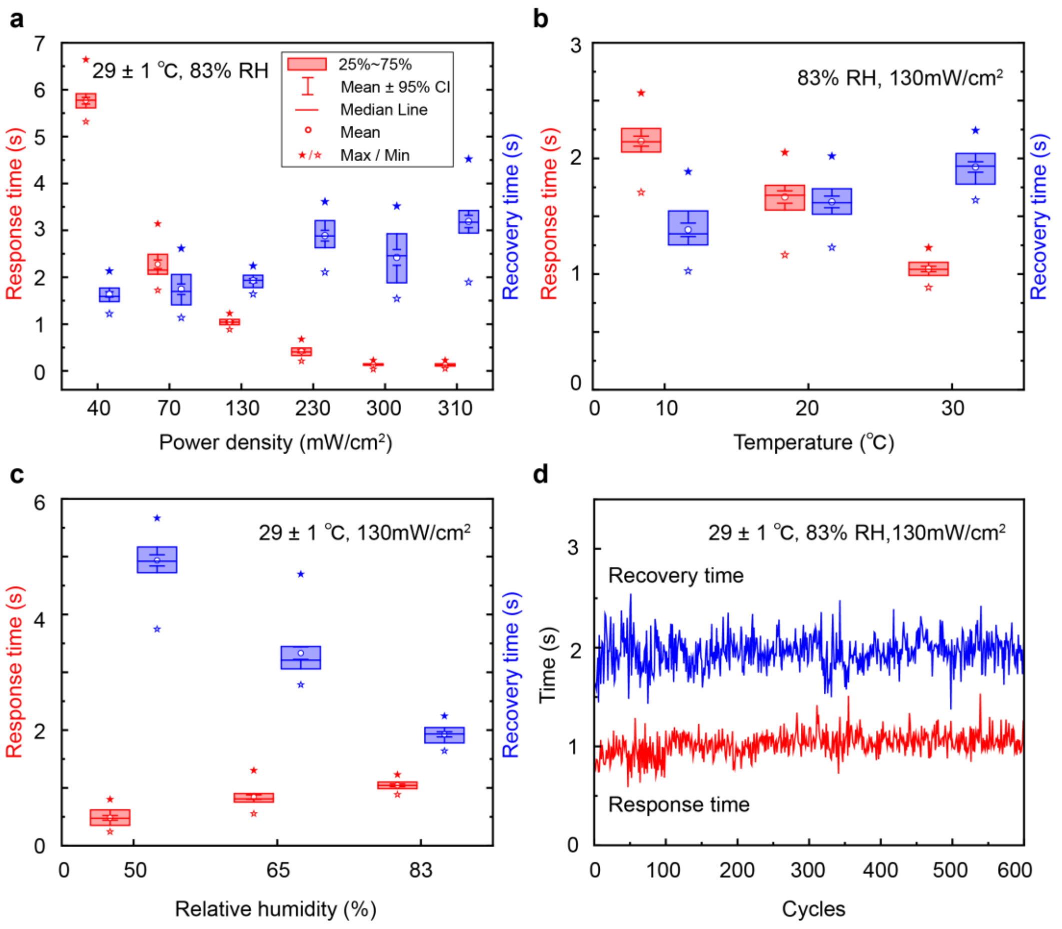

Supplementary Fig. 6. Environmental resilience and cycling stability of the single-pole single-throw (SPST) relay. **a** Response and recovery times as a function of light intensity (40–310 mW/cm^2) at 29 ± 1 °C and 83 ± 3% RH. **b** Effect of ambient temperature (10–30 °C) on response and recovery time under 130 mW/cm^2 and 83 ± 3% RH. **c** Influence of relative humidity (50–83% RH) on response and recovery time under 29 ± 1 °C and 130 mW/cm^2. The red box-and-whisker plot represents the response time, while the blue box-and-whisker plot represents the recovery time. Each dataset (a-c) is based on 50 measurements for the respective conditions (n=50). The filled star, hollow star, circles, boxes, lines in the box, and error bars represent maximum values, minimum values, mean values, 25–75% distributions, median values, and 95% confidence interval (CI), respectively. **d** Cycling endurance test at 130 mW/cm^2, 29 ± 1 °C, and 83 ± 3% RH, showing stable operation over 600 cycles.

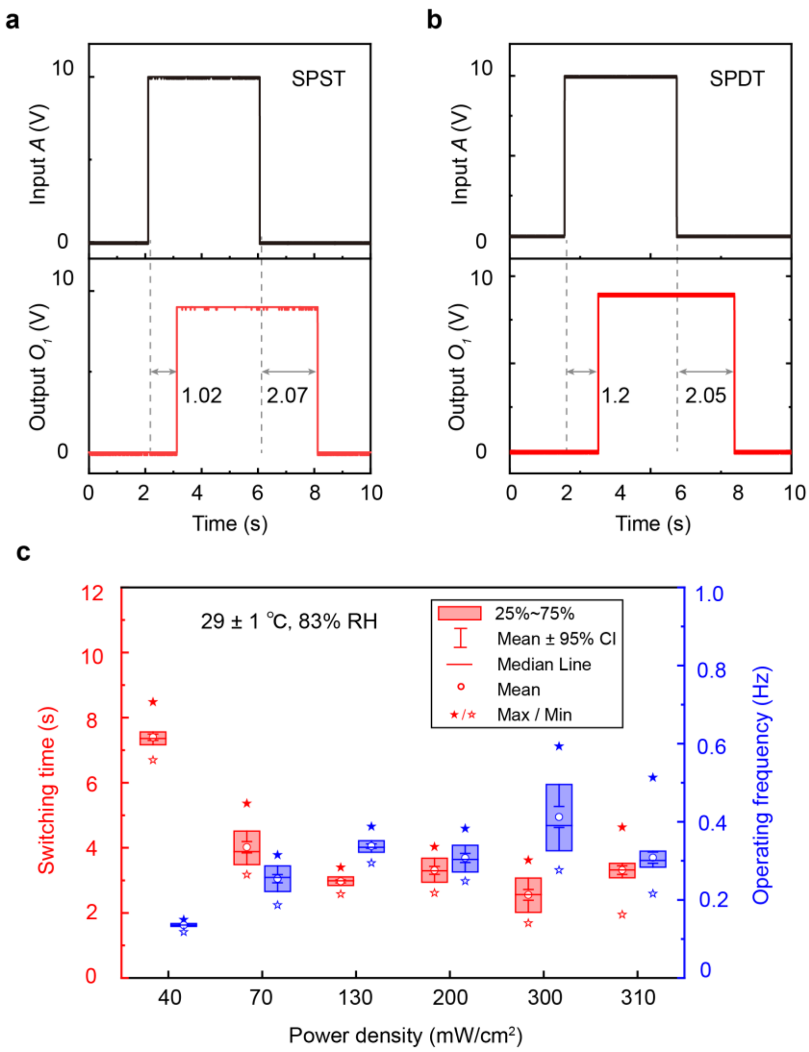

Supplementary Fig. 7. Comparison of single-pole single-throw (SPST) and single-pole double-throw relays (SPDT) relay performance, and environmental effects on switching performance of the SPST relay. a-b Response and recovery times of the SPST and SPDT relays under illumination of 130 mW/cm^2 at 29 ± 1 °C and 83 ± 3 % RH. **c** Switching times and operating frequency of the SPST relay as a function of light intensity (40–310 mW/cm^2) at 29 ± 1 °C and 83 ± 3% RH. The red box-and-whisker plot represents the response time, while the blue box-and-whisker plot represents the recovery time. Each dataset is based on 50 measurements for the respective conditions (n=50). The filled star, hollow star, circles, boxes, lines in the box, and error bars represent maximum values, minimum values, mean values, 25–75% distributions, median values, and 95% confidence interval (CI), respectively.

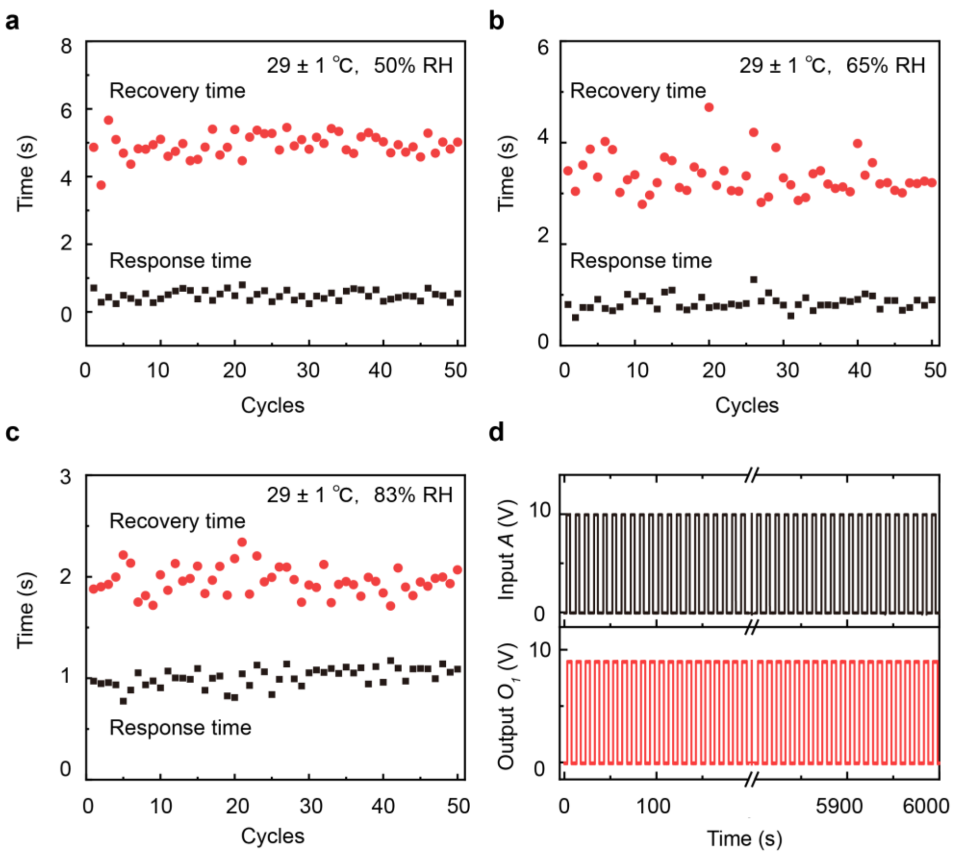

Supplementary Fig. 8. Humidity-assisted reversibility and cycling stability of the single-pole single-throw (SPST) relay. a–c Response and recovery dynamics at different relative humidity levels for 50 cycles (50 ± 3%RH, 65 ± 3%, and 83 ± 3% RH). **d** Cycling endurance over 600 actuation–recovery cycles under illumination of 130 mW/cm^2 at 29 ±1°C and 83 ± 3% RH.

Supplementary Note 2. Numerical simulation of photothermal–mechanical–electrical conversion process of SPST relay

We developed a finite element simulation framework to quantitatively model the photothermal–mechanical–electrical conversion process in our SPST relay. The model integrates two key components: (1) a heat transfer module to capture the spatial–temporal evolution of the temperature field under optical illumination, and (2) a thermo-mechanical deformation module based on beam theory to describe light-induced bending and electrode contact. For simplicity, the intermediate microlayer (Ag NWs layer) was neglected in the simulations since its thickness is more than an order of magnitude smaller than that of the upper and lower films (The thicknesses of the PDMS/Ag NWs/PANi-PNIPAm film are 70 μm, 5 μm, and 134 μm, respectively).

(1) Heat Transfer Model

The optical absorption and heat transport were simulated by coupling the *Radiation in Absorbing Media* and *Heat Transfer in Solids* modules. Optical energy was converted into a volumetric heat source via the Beer–Lambert law, and the transient heat conduction equation was solved:

$$\rho C_p \frac{\partial T}{\partial \mathrm{t}} + \nabla \cdot (-k \nabla T) = Q \tag{1}$$

where ρ, C_{p}, and k are the density, specific heat capacity, and thermal conductivity, respectively.

The volumetric heat source Q is determined by

$$Q = \eta \mu_a I \tag{2}$$

where μ_a is the optical absorption coefficient, I is the light intensity, and η is the photothermal conversion efficiency.

(2) Thermo-Mechanical Deformation Model

The thermally induced bending was modeled using a fixed-end cantilever beam approximation. The beam is treated as an elastic solid, whose deformation and stress response follow solid mechanics theory. The governing equations consist of the equilibrium equation (momentum conservation), the geometric relation (strain–displacement relation), and the constitutive relation (stress–strain relation with thermal effects). The deformation follows linear elasticity theory, governed by the Equilibrium equation:

$$\nabla \cdot \boldsymbol{\sigma} + \mathbf{F}_v = \rho \frac{\partial^2 \mathbf{u}}{\partial t^2} \tag{3}$$

Where $\boldsymbol{\sigma}$ is the stress tensor, $\boldsymbol{F}_v$ is the body force vector (neglected in this model), ρ is the material density, and $\boldsymbol{u}$ is the displacement vector (m).

Considering small deformations, the small-strain assumption is adopted and the strain-displacement relation:

$$\boldsymbol{\epsilon} = \frac{1}{2}[\nabla\mathbf{u} + (\nabla\mathbf{u})^{\mathrm{T}}] \tag{4}$$

where $\boldsymbol{\epsilon}$ is the small strain tensor (dimensionless). The stress–strain relation under thermoelastic deformation is written as:

$$\boldsymbol{\sigma} = \mathbf{C} : (\boldsymbol{\epsilon} - \boldsymbol{\epsilon}_{th}) \tag{5}$$

where $\mathbf{C}$ is the elastic stiffness tensor, which for isotropic materials is characterized by the Young's modulus E and Poisson's ratio ν. $\boldsymbol{\epsilon}_{th}$ is the thermal strain tensor (dimensionless).

The thermal strain is given by:

$$\boldsymbol{\epsilon}_{th} = \alpha\left(T - T_{ref}\right)\boldsymbol{I} \tag{6}$$

Here, α is the coefficient of thermal expansion, T is the current temperature, T_{ref} is the reference temperature (material's initial temperature), and $\mathbf{I}$ is the identity tensor.

(3) Modeling Parameters

PDMS: Elastic modulus = 2 MPa; Poisson's ratio = 0.49; thermal conductivity = 0.15 $W \cdot m^{-1} \cdot K^{-1}$; density = 970 $kg \cdot m^{-3}$; specific heat capacity = 1460 $J \cdot kg^{-1} \cdot K^{-1}$; coefficient of thermal expansion = 5.67×10^{-4} K^{-1}.

PANi–PNIPAm: Elastic modulus = 1 MPa; Poisson's ratio = 0.49; thermal conductivity = 0.6 $W \cdot m^{-1} \cdot K^{-1}$; density = 1200 $kg \cdot m^{-3}$; specific heat capacity = 2100 $J \cdot kg^{-1} \cdot K^{-1}$; coefficient of thermal expansion = 0 below 32 °C and -1×10^{-6} K^{-1} above 32 °C.

Based on these parameters, finite element simulations were conducted to model the coupled photo–thermal–mechanical–electrical transformation process in the relay, capturing the temperature evolution, induced deformation, and subsequent electrical contact behavior.

Simulations show that under 130 mW/cm^2 illumination, the polyaniline composite film (PCF) temperature exceeded the LCST within 0.05 s and reached ~45.7 °C at 1 s (Supplementary Fig. 9a (i-iii)). The transient temperature rise at the PCF center is presented in Supplementary Fig. 9c.

The simulated bending profile under 130 mW/cm^2 illumination at t = 1 s is shown in Supplementary Fig. 9b, and the corresponding dynamic displacement of the beam end is provided in Fig. 9b (i-iii). The time-dependent displacement curve in Supplementary Fig. 9d further confirms that electrode contact occurs after approximately 1 s. Both experimental and simulation results show a strong dependence of response time on illumination intensity (Supplementary Fig. 10).

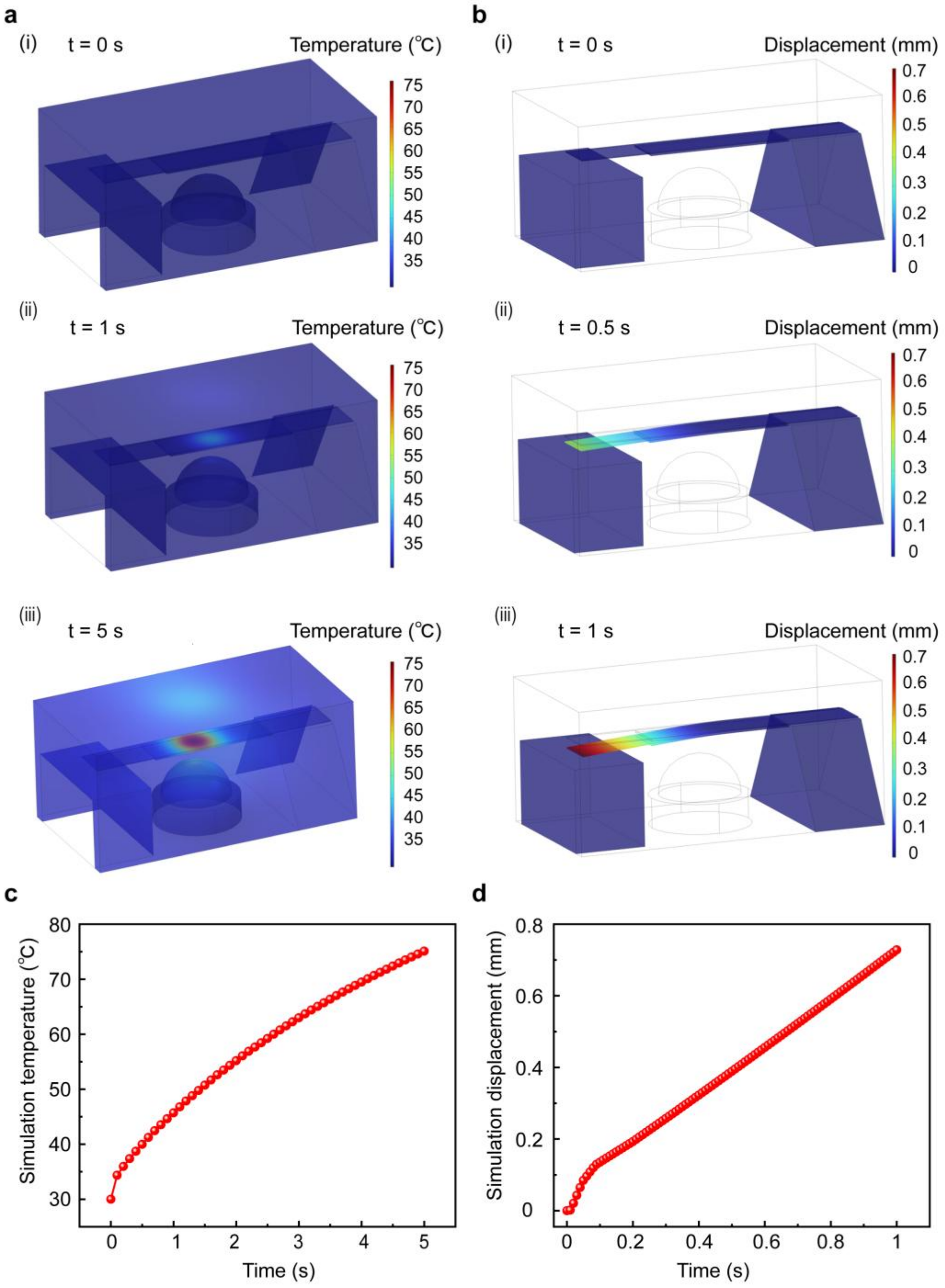

Supplementary Fig. 9. The simulation results of Heat Transfer Model and Thermo-Mechanical Deformation Model. a Simulated temperature distribution across the suspended polyaniline composite film (PCF) at t = 0， 1， 5 s under optical illumination (130 mW/cm^2). **b** Simulated displacement distribution of the beam end at t = 0，0.5，1 s under optical illumination (130 mW/cm^2), indicating bending deformation. **c** Transient evolution of the center-point temperature of the PCF, showing heating dynamics under 130 mW/cm^2 illumination. **d** Time-dependent displacement of the beam end under 130 mW/cm^2 illumination, where contact with the electrode occurs after ~1 s.

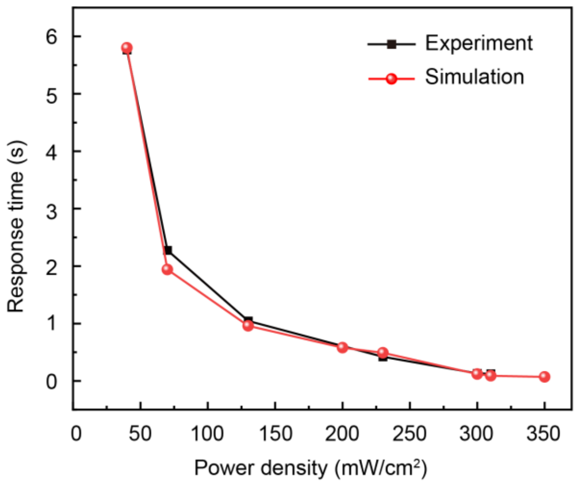

Supplementary Fig. 10. **Simulated and experimental results of response time as a function of input light power density.**

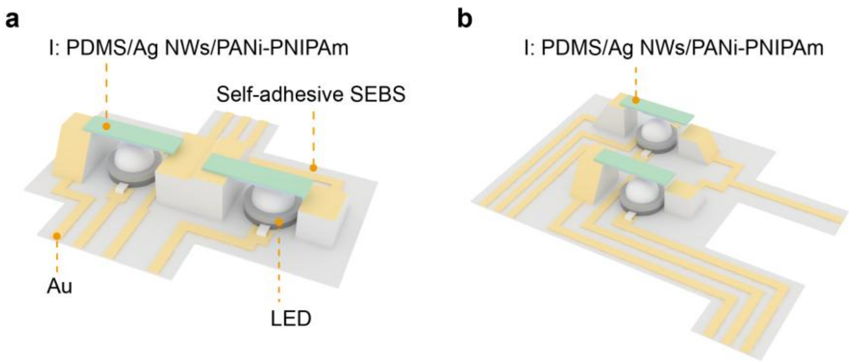

Supplementary Fig. 11. Schematic of logic gate. **a** AND gate, **b** OR gate. The AND and OR gates are realized by connecting two single-pole single-throw (SPST) relays in series and parallel, respectively. Each SPST relay consists of a polyaniline composite film (PCF) I (PDMS/Ag NWs/PANi-PNIPAm).

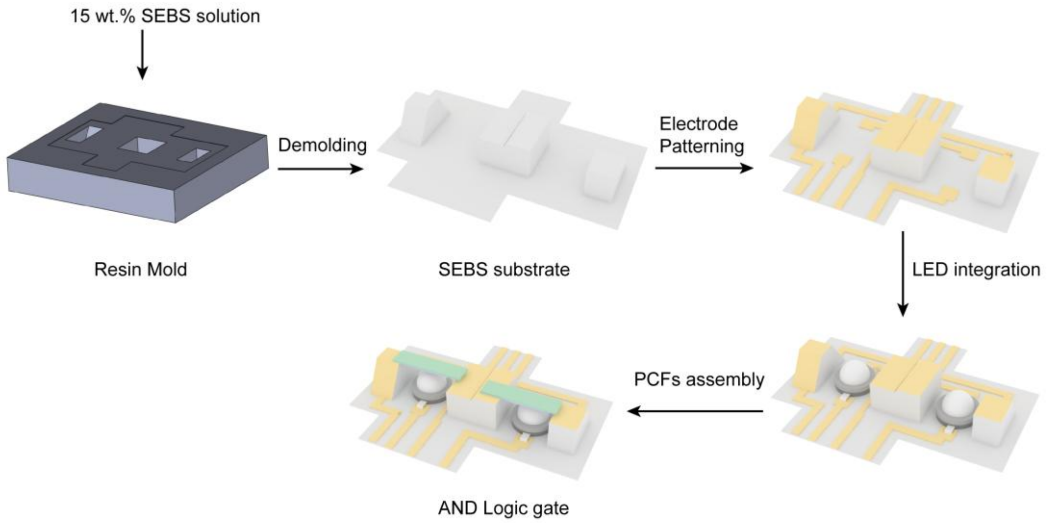

Supplementary Fig. 12. Fabrication process flow for AND logic gate. (i) Shape formation: Mold-casting of styrene-ethylene-butylene-styrene (SEBS)/toluene solution followed into predefined architectures. (ii) Demolding: Demolding to obtain free-standing SEBS substrates. (iii) Electrode patterning: Au deposition via e-beam evaporation with shadow masking. (iv) Bulb integration: Bulb mounting using Ag paste and double-sided adhesive tape. (v) Polyaniline composite film (PCF) assembly: Oriented attachment of PCF (PDMS/AgNWs/PANi-PNIPAm) to Au electrodes.

Supplementary Note 3. Self-adhesive SEBS Connections for Instant Electrical Bonding

The optical images of two types of self-adhesive styrene-ethylene-butylene-styrene (SEBS) connections are shown in Supplementary Fig. 13. To validate the logical functionality, we designed polyimide (PI) flexible circuit boards (FCBs), as illustrated in Supplementary Fig. 14. The self-adhesive SEBS connections exhibit intrinsic self-adhesive properties, facilitating the rapid establishment of electrical connections between functional interfaces. To establish an electrical connection between devices A and B using SEBS connections, the ends of the SEBS connections are aligned with the electrode pads of A and B, respectively. The aligned sections are then manually compressed for 5–10 seconds, ensuring the rapid formation of a reliable electrical bond between A and B, without the need for adhesives or thermal processing. This bonding process is demonstrated in two scenarios: (i) SEBS connection between logic gates and PI FCB (Supplementary Movie 4), and (ii) SEBS connection between interconnected logic gate arrays (Supplementary Movie 7).

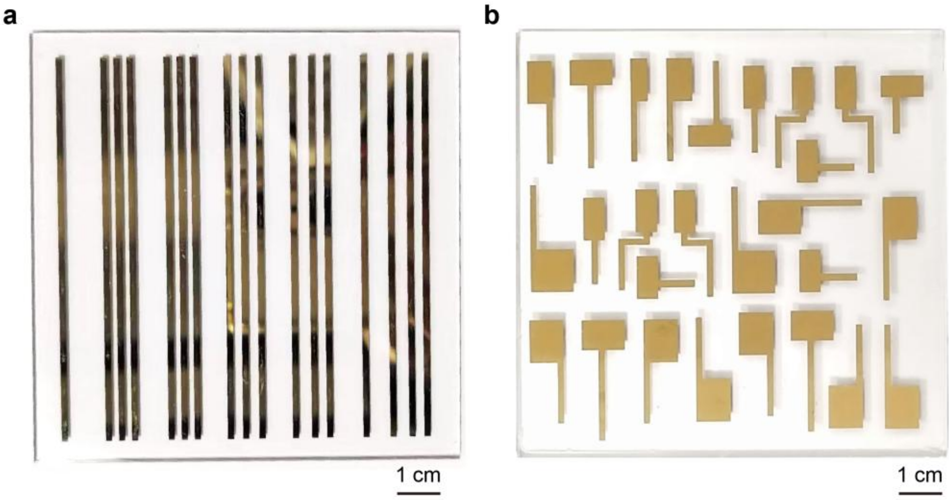

Supplementary Fig. 13. The self-adhesive styrene-ethylene-butylene-styrene (SEBS) connections: with patterned gold electrodes for flexible hybrid electronics. **a** SEBS connections employed for logic gate-to-logic gate interfaces. Scale bar: 1 cm. **b** SEBS connections employed for logic gate-to- polyimide flexible circuit board (PI FPC) interfaces. The SEBS connections are custom-trimmed to match specific interface geometries during relay integration. Scale bar: 1 cm.

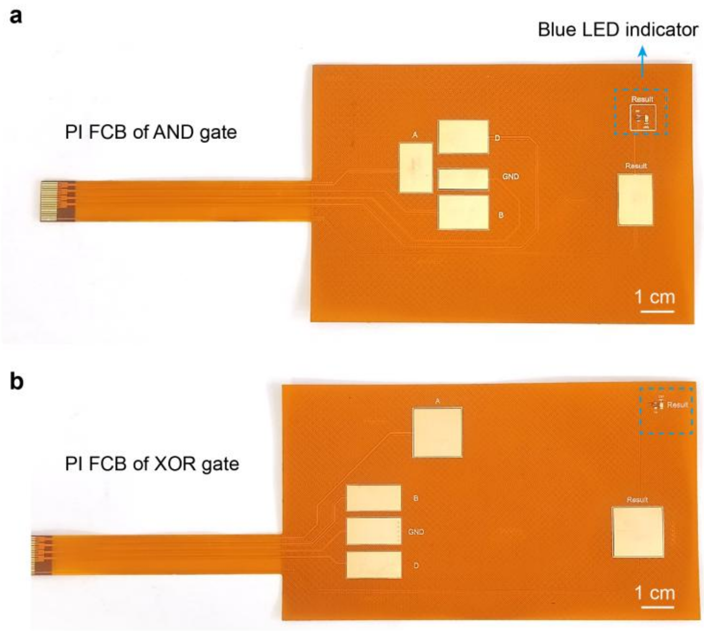

Supplementary Fig. 14. Polyimide flexible circuit board (PI FCB) configurations for logic gate evaluation. a AND gate, **b** XOR gate. The blue LED visualizes the output states. Scale bar: 1 cm for all images.

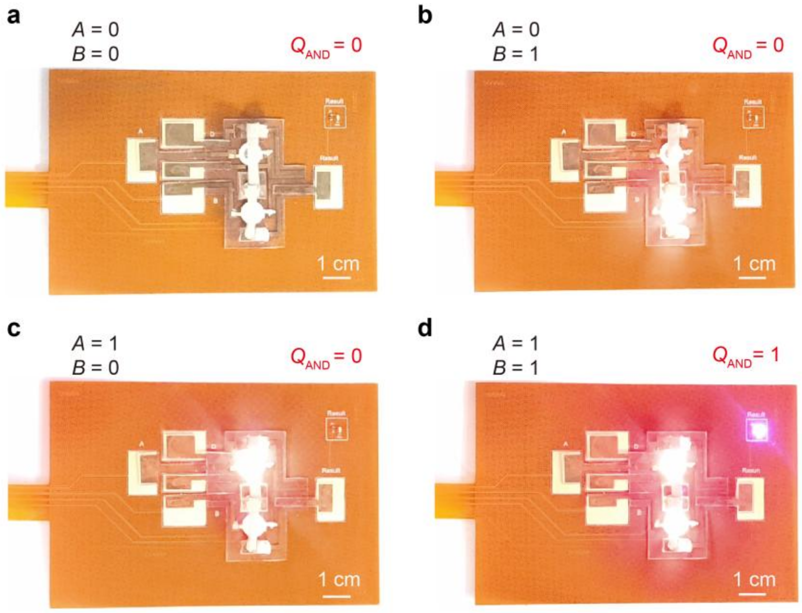

Supplementary Fig. 15. Experimental images of the AND logic gate under combinatorial inputs. a-d Truth table validation: **a** $A = 0$, $B = 0$. **b** $A = 0$, $B = 1$. **c** $A = 0$, $B = 1$. **d** $A = 1$, $B = 1$. Output states are optically indicated by a blue LED (ON=logic 1, OFF=logic 0). Scale bar: 1 cm for all images.

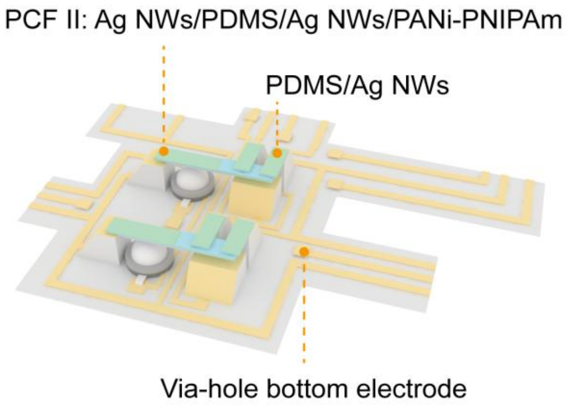

Supplementary Fig. 16. Schematic representation of the XOR logic gate. The XOR gate is realized by connecting two single-pole double-throw (SPDT) relays in parallel. Each SPDT relay consists of a polyaniline composite film (PCF) II (Ag NWs/PDMS/Ag NWs/PANi-PNIPAm) and two PDMS/Ag NWs. The gate is equipped with square via-hole bottom electrodes to prevent short-circuiting.

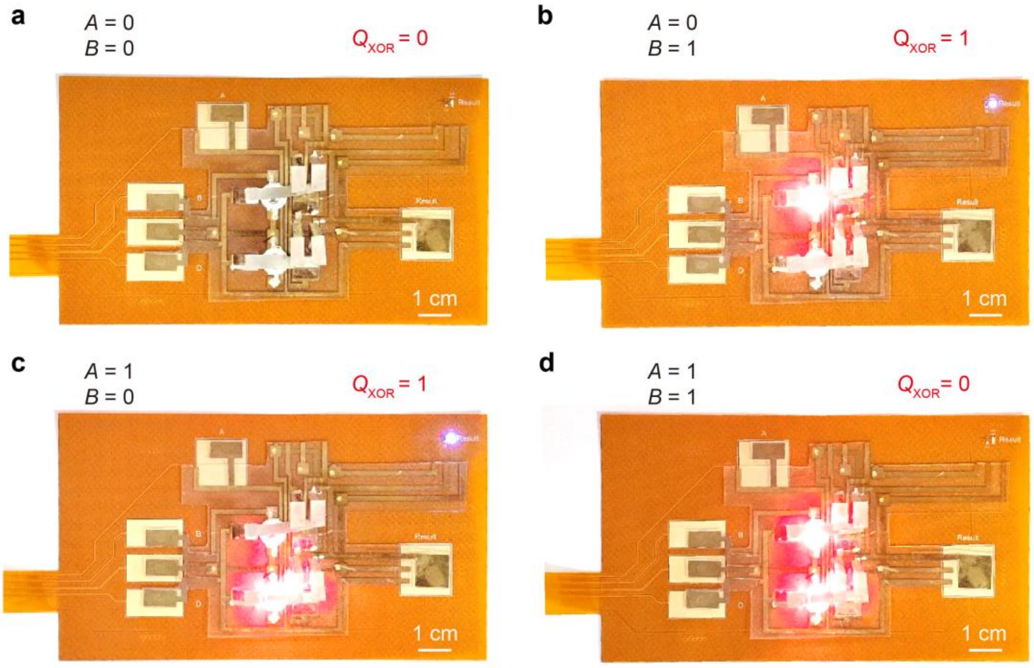

Supplementary Fig. 17. Experimental images of XOR logic gate under combinatorial inputs. a-d Truth table validation: **a** $A = 0$, $B = 0$. **b** $A = 0$, $B = 1$. **c** $A = 1$, $B = 0$. **d** $A = 1$, $B = 1$. Output states are optically indicated by a blue LED (ON=logic 1, OFF=logic 0). Scale bar: 1 cm for all images.

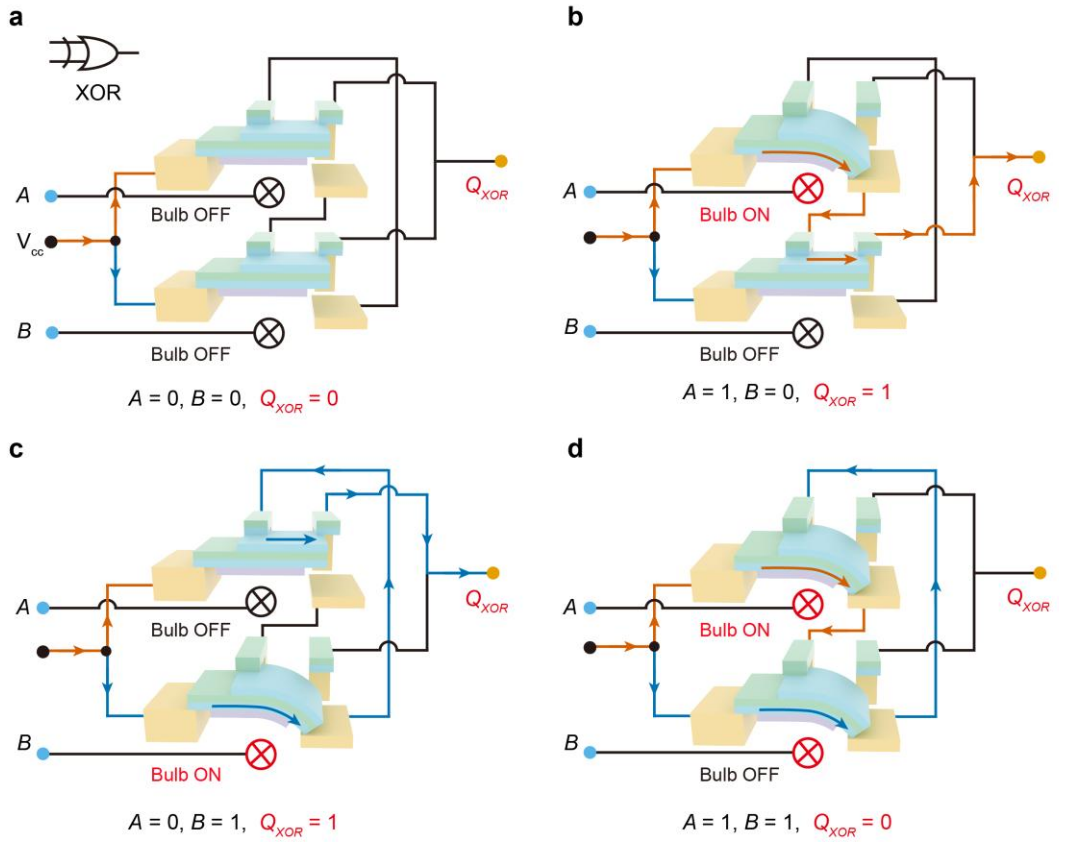

Supplementary Fig. 18. Schematic of the operational mechanism of XOR gate. Panels **a–d** show relay states, the flow of electrical signals, and logic output for the four possible input combinations, with vermilion and blue arrows indicating signal direction. The optical inputs (*A*, *B*) are indicated in sky blue solid circles, the electrical output (Q_{XOR}) in orange solid circles, and the input voltage (V_{cc}) in larger black solid circles. The black and red bulb symbols denote the light source in the off and on states, respectively.

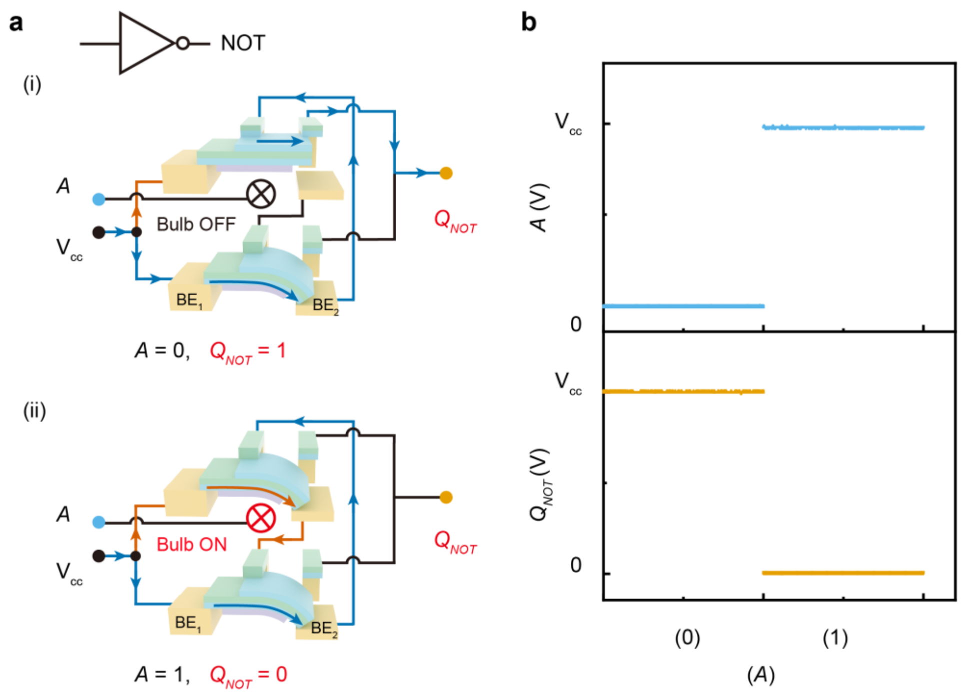

Supplementary Fig. 19. The realization of NOT gate. **a** Schematic of NOT gate realized by an XOR, with the bottom polyaniline composite film (PCF) connected and fixed to BE_1 and BE_2 via silver conductive paint, leaving *A* as the sole variable input. Panels (i)-(ii) show relay states, the flow of electrical signals, and logic output for the two possible input combinations, with vermilion and blue arrows indicating signal direction. The optical input *A* are indicated in sky blue solid circles, the electrical output (Q_{NOT}) in orange solid circles, and the input voltage (V_{cc}) in larger black solid circles. **b** Measured input (*A*) and output (Q_{NOT}) voltage characteristics. The black and red bulb symbols denote the light source in the off and on states, respectively.

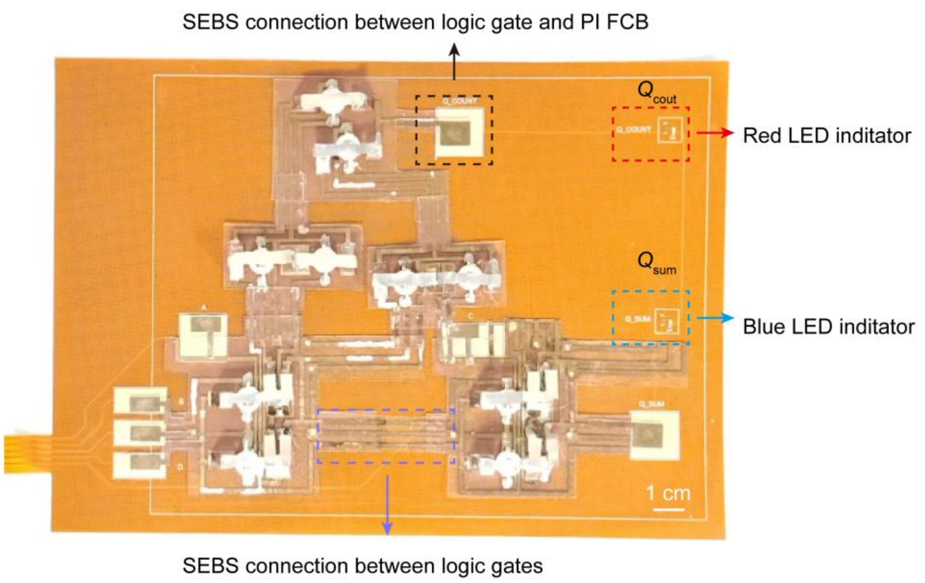

Supplementary Fig. 20. The polyimide flexible circuit board (PI FCB) used for demonstrating the full-adder operation. Electrical bonding is established through two types of styrene-ethylene-butylene-styrene (SEBS) connections: SEBS connection between the logic gate and PI FCB, and SEBS connection between logic gates. Blue and red LEDs, integrated into the PI FCB, visualize the output states by indicating Q_{Sum} and Q_{Cout}, respectively. Scale bar: 1 cm.

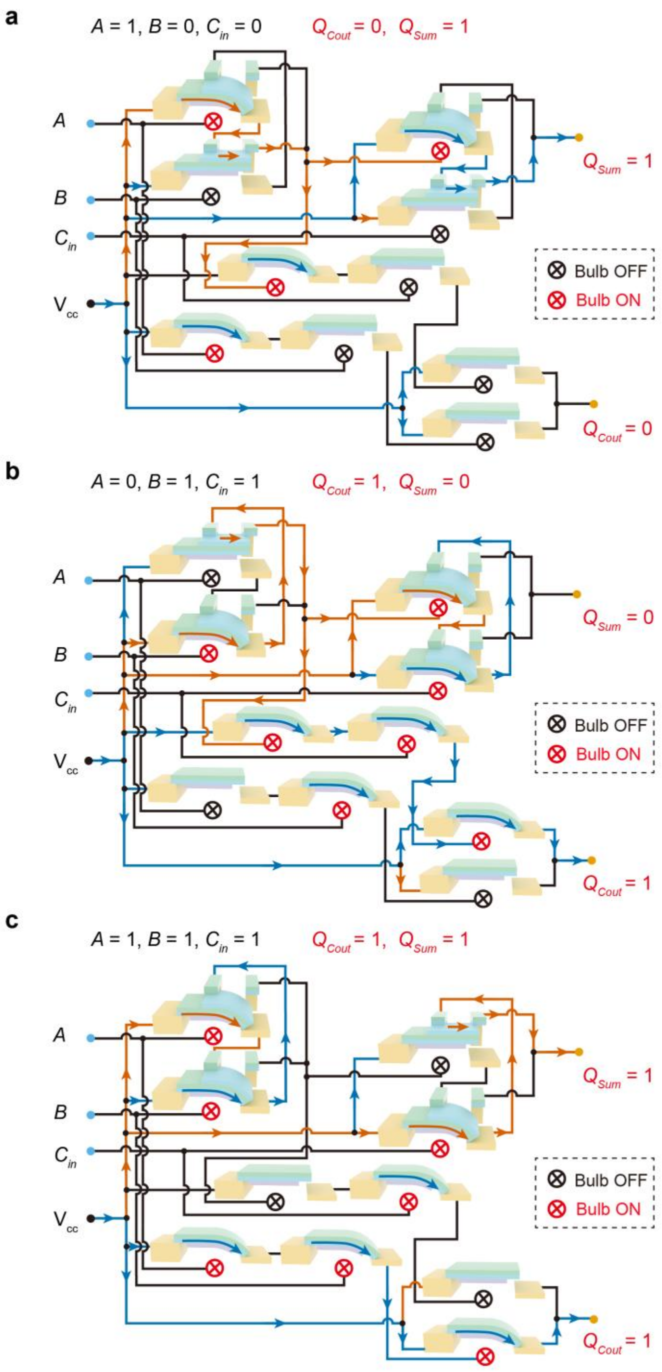

Supplementary Fig. 21. Schematic of operational mechanism of the 1-bit full-adder circuit. Panels **a–c** show relay states, the flow of electrical signals, and logic output for the three possible input combinations, with vermilion and blue arrows indicating signal direction. The optical input *A* are indicated in sky blue solid circles, the electrical output (Q_{Sum}, Q_{Cout}) in orange solid circles, and the input voltage (V_{cc}) in larger black solid circles. The black and red bulb symbols denote the light source in the off and on states, respectively.

Supplementary Note 4: Scalability Assessment

The scalability of the light-programmable relay system is essential for advancing its application to more complex logical operations. To evaluate this, we demonstrate the realization of a 2-bit adder, showcasing the system's capacity to scale to more sophisticated logic functions. As shown in Supplementary Fig. 22a, the logic diagram of the 2-bit adder comprises four inputs (A_1, B_1, A_2, B_2) and three outputs (Q_{cout}, Q_{S2}, Q_{S1}). The corresponding experimental setup and truth table are presented in Supplementary Fig. 22b–c. Fig. 22d shows the experimentally measured outputs for all 16 input combinations, which match the truth table with complete accuracy. These results confirm the scalability of the light-driven mechanical logic system and provide experimental evidence and design insights for realizing larger-scale, general-purpose light-responsive computing materials.

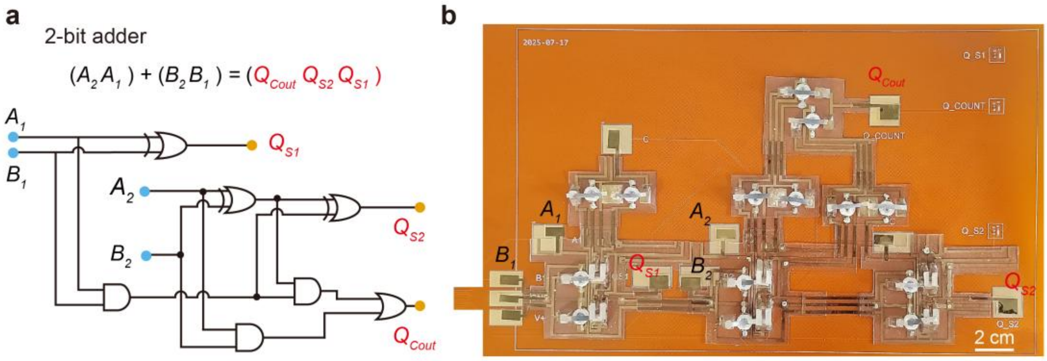

c

A_1	B_1	A_2	B_2	Q_{Cout}	Q_{S2}	Q_{S1}
1	1	1	1	1	1	0
0	1	1	0	0	1	1
1	1	1	0	1	0	0
1	0	1	1	1	0	1
0	0	1	0	0	1	0
0	1	1	1	1	0	1
0	0	0	1	0	1	0
1	1	0	1	1	0	0
1	0	1	0	0	1	1
0	0	1	1	1	0	0
1	0	0	1	0	1	1
0	0	0	0	0	0	0
0	1	0	1	0	1	1
1	0	0	0	0	0	1
1	1	0	0	0	1	0
0	1	0	0	0	0	1

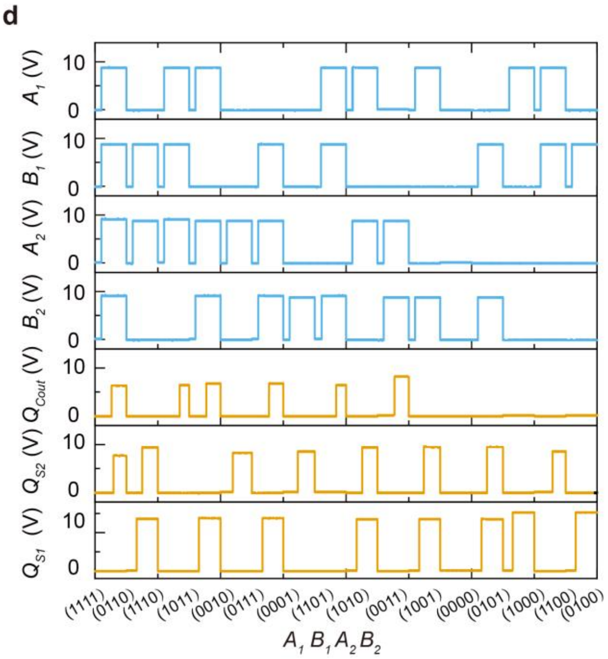

Supplementary Fig. 22. Demonstration of a 2-bit full adder. **a** Schematic of the 2-bit adder, showing four inputs (A_1, B_1, A_2, B_2) in sky blue and three outputs (Q_{cout}, Q_{S2}, Q_{S1}) in orange. **b** Experimental setup of the 2-bit adder comprising three XOR gates, three AND gates, and one OR gate. The scale bar is 1cm. **c** Truth table for all 16 possible input combinations. **d** Measured input voltages and output results for all 16 input combinations.

Supplementary Note 5. Humidity control system

To address humidity-assisted resetting. we designed and demonstrated an autonomous control system. Specifically, a NOR gate was integrated into the input section of signals *A* and *B*: when both inputs are 0 corresponding to the absence of light signals, the humidifier is automatically activated to reset the system; when either input is 1 indicating active computation, the humidifier remains off to avoid interfering with logic operations.

As shown in Supplementary Fig. 23a (schematic) and Supplementary Fig. 23b (photograph), this self-regulated reset strategy allows the relay to autonomously control its recovery process. Supplementary Movie 9 further illustrates the operation of this mechanism during an AND gate computation, where the system successfully resets without manual intervention. This design provides a practical pathway toward autonomous control, thereby enhancing the real-world applicability of our light-programmable mechanical computing platform.

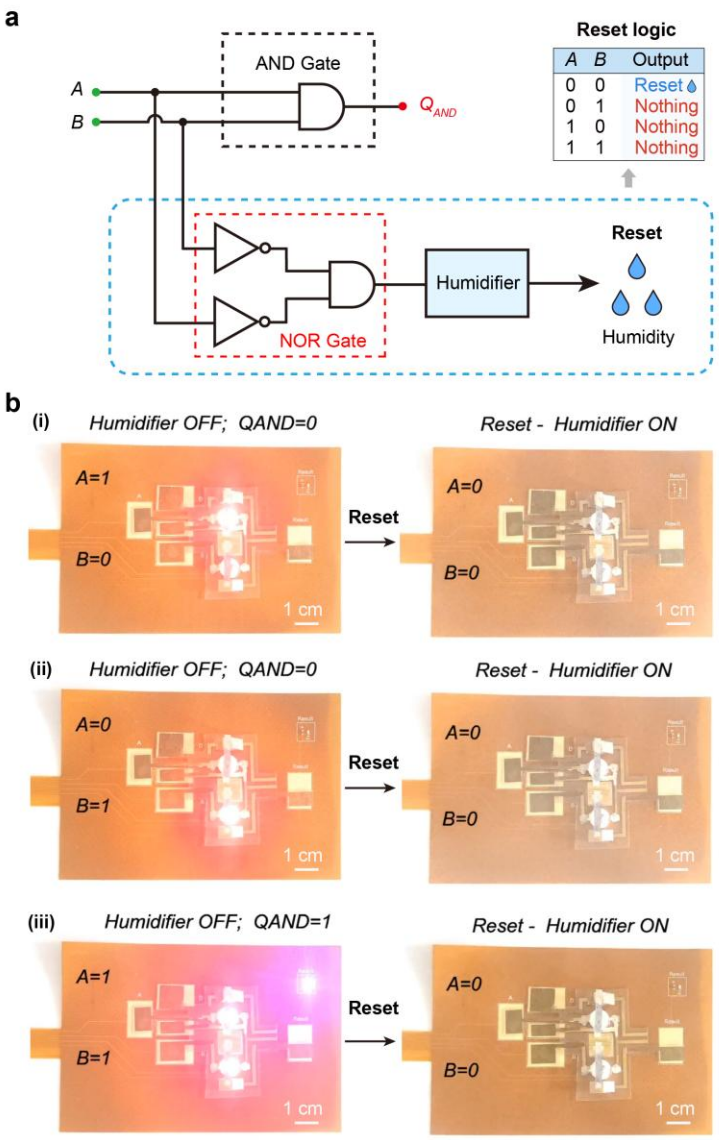

Supplementary Fig. 23. Humidity control system. a Schematic of the humidity control system. A NOR gate is introduced to autonomously activate the humidifier when both input signals (A, B) are 0, enabling system reset. **b** Photographs showing the system reset process when input combinations (1,0), (0,1), and (1,1) transition to (0,0), demonstrating the effectiveness of the autonomously controlled resetting mechanism. Scale bar: 1 cm for all images.

Supplementary Note 6. The Realization of Adaptive Texture Camouflage

(1) The actual computational capability

To evaluate the system's computational performance in real-world scenarios, we constructed a physical demonstration platform consisting of a 3 × 3 unit, as illustrated in Supplementary Fig. 24a. Each sensing-computation-emission (SCE) unit integrates light perception, logical computation, and pattern reconstruction functionalities. The system was experimentally tested to reproduce texture camouflage patterns. Supplementary Figs. 24b, d, and f show the original input images and their corresponding schematic camouflage patterns generated by the computing circuit, while Supplementary Figs. 24c, e, and g present the experimental results. A demonstration video (Supplementary Movie 10) further provides direct visual confirmation that the experimentally reconstructed camouflage images closely match the simulated results. These results validate the operational capability of the basic units and confirm that the light-programmable computing system can successfully execute texture reconstruction in real-world conditions.

(2) Image texture similarity evaluation method

We quantitatively assessed image texture similarity using a multi-feature fusion strategy. Images were first preprocessed via grayscale conversion, size alignment, and normalization to ensure comparability. Complementary descriptors were then extracted from both spatial and frequency domains. In the spatial domain, gray-level co-occurrence matrices (GLCMs) provided contrast, correlation, energy, and homogeneity metrics, while local binary pattern (LBP) histograms captured fine-scale microstructures. In the frequency domain, fast Fourier transforms (FFT) yielded radial power spectral density and angular energy distribution, from which an anisotropy index characterized directional preferences. The Wiener–Khinchin theorem was applied to estimate the autocorrelation function, revealing periodic structures, and structure tensor coherence quantified the directional order of local gradients. Each descriptor was normalized to [0,1], and a weighted average produced the final composite similarity score. By integrating local statistics, spectral features, and directional order, this approach robustly distinguishes structured textures from stochastic noise, providing a reliable measure of image similarity.

(3) The response error of the system under complex environment

We analyzed the system's robustness when exposed to environmental disturbances, such as variations in temperature, humidity, and mechanical stress. These factors may induce local failure in individual units, potentially leading to errors in the camouflage output. To assess this effect, environmental perturbations were modeled as random damage to a given proportion of functional units, which consequently lose their emissive capability. Due to the distributed design of the SCE framework, such local failures do not propagate across the system, thereby preserving global functionality. To quantitatively evaluate this robustness, we simulated camouflage performance under increasing levels of unit damage and analyzed image fidelity using a comprehensive texture similarity metric that integrates spatial and frequency-domain descriptors (Supplementary Fig. 25). The analysis reveals that even when a substantial fraction of units becomes nonfunctional, the camouflage images maintain high structural similarity to the target textures. This result highlights the intrinsic fault tolerance and resilience of the proposed design.

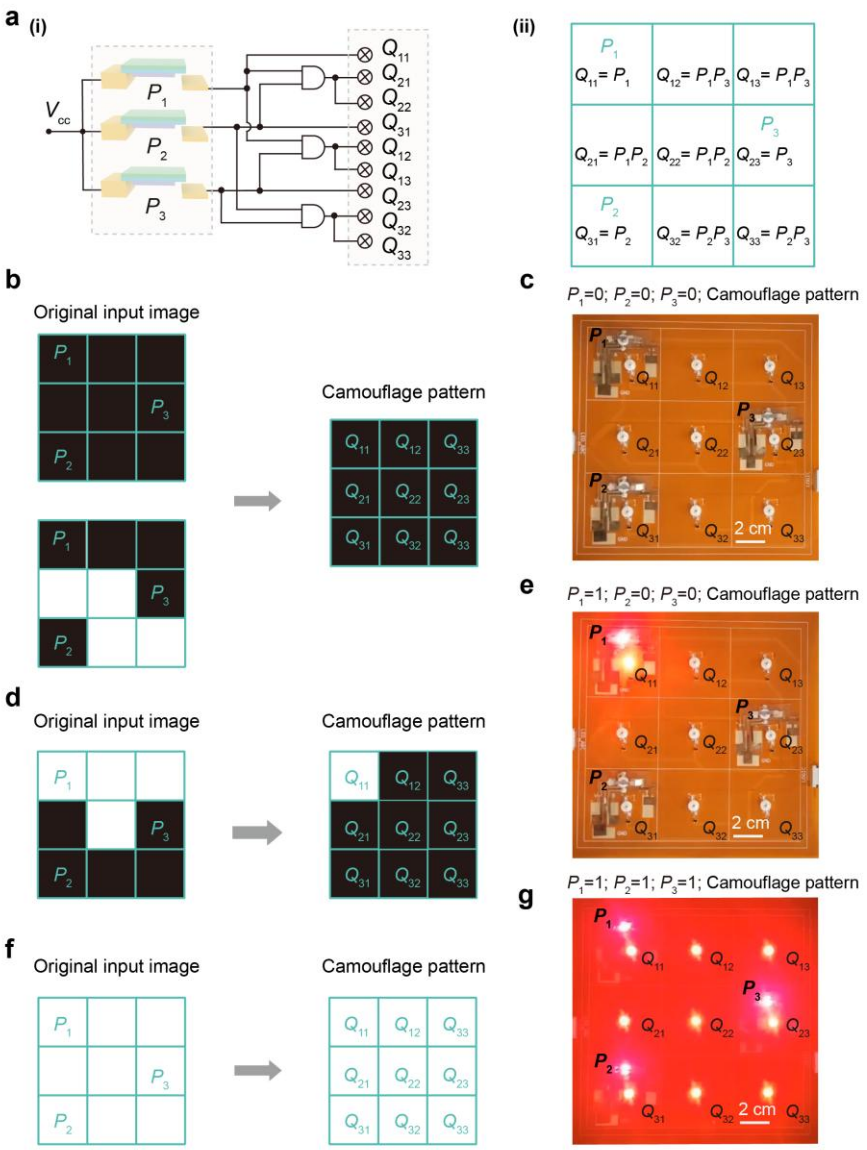

Supplementary Fig. 24. Experimental validation of the texture camouflage reconstruction unit. a (i) sensing–computation–emission (SCE) unit, (ii) The spatial distribution of the sensed optical input signals (P_1, P_2, and P_3) and the corresponding output signals (Q_{11}, Q_{12}, …, Q_{33}) in the SCE unit, as well as the logical relationships between the input and output signals. **b, d, f** Schematic illustrations of the original input images and the corresponding camouflage patterns generated by the computing circuit. **c** Experimental result corresponding to the camouflage pattern shown in **b**, where all pixels within the 3 × 3 unit remain in the off state. Scale bar: 2 cm. **e** Experimental result corresponding to the camouflage pattern shown in **d**, where only the upper-left pixel within the 3 × 3 unit is in the on state. Scale bar: 2 cm. **g** Experimental result corresponding to the camouflage pattern shown in **e**, where all pixels within the 3×3 unit are in the on state. Scale bar: 2 cm.

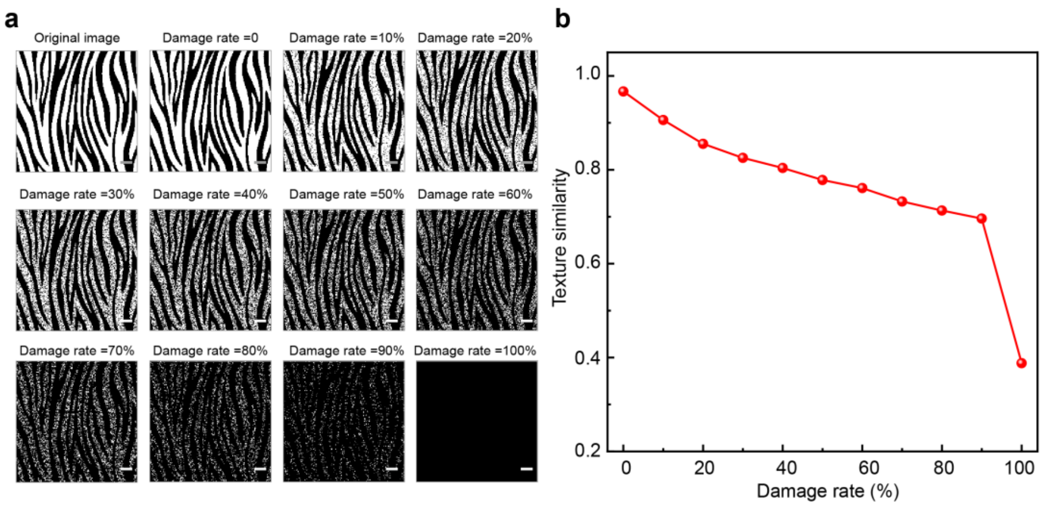

Supplementary Fig. 25. Robustness characteristics of the camouflage system. a Comparison between original image and camouflaged outputs as the proportion of randomly damaged unit structures increases from 0% to 100%. The image resolution is 1024 × 1024. Scale bar:100 pixels. **b** Quantitative analysis of image similarity between original and generated images at different damage levels, with the x-axis representing damage proportion and the y-axis showing texture similarity.

Supplementary Note 7. The achievable texture reconstruction fidelity and the expressive capability of the camouflage system

(1) Discuss how this reduction in independent output limits the expressibility and achievable fidelity of the camouflage pattern

Overall impact on expressibility and achievable fidelity

In our system, the representational capacity and achievable fidelity of the camouflage patterns are primarily governed by the relationship between the characteristic feature size of the target texture and the spatial sampling area of a single sensing-computation-emission (SCE) unit (3×3 pixels). When the size of texture features is substantially larger than the sensing window size, the reconstructed patterns maintain global continuity and exhibit high visual fidelity. In contrast, when the feature size approaches the 3×3 sensing scale, the unit can no longer resolve the underlying texture variations, leading to reduced reconstruction accuracy and diminished expressive capability.

Underlying mechanism of the camouflage pattern

This behavior arises directly from the operating principle of each SCE unit. Each unit samples local intensity variations by using three spatially distributed sensing nodes and reconstructs a representative local gradient direction through an AND-based logic pathway, as shown in Supplementary Fig. 26a. This mechanism yields a clean abstraction of texture features by emphasizing coarse spatial trends such as prominent edges and stripe boundaries, while intentionally omitting fine-scale variations within a 3×3 region. Accordingly, the reconstruction fidelity is determined by the relationship between the texture feature size and the spatial sampling window of the SCE unit. When the texture features extend beyond the sampling region, the unit effectively captures the underlying gradient variations and consequently achieves high-fidelity reconstruction of local texture patterns.

Validation of the fidelity of the camouflage patterns

To further quantify this effect, we carried out systematic simulations using zebra-stripe textures with different feature sizes, with the corresponding results shown in Supplementary Fig. 27a. Reconstruction quality was assessed using the pixel mismatch rate between the input and reconstructed images, defined as the ratio of pixels with inconsistent brightness to the total number of pixels (Supplementary Fig. 28). When the average stripe width in the input image exceeds approximately four mapped pixels, the

mismatch rate remains below 15%, indicating that the system maintains camouflage-level fidelity. As the stripe width further decreased to approximately 2 pixels, approaching the scale of the 3 × 3 sensing window, a reduction in reconstruction fidelity emerged. This behavior is fully consistent with the expected operating mechanism.

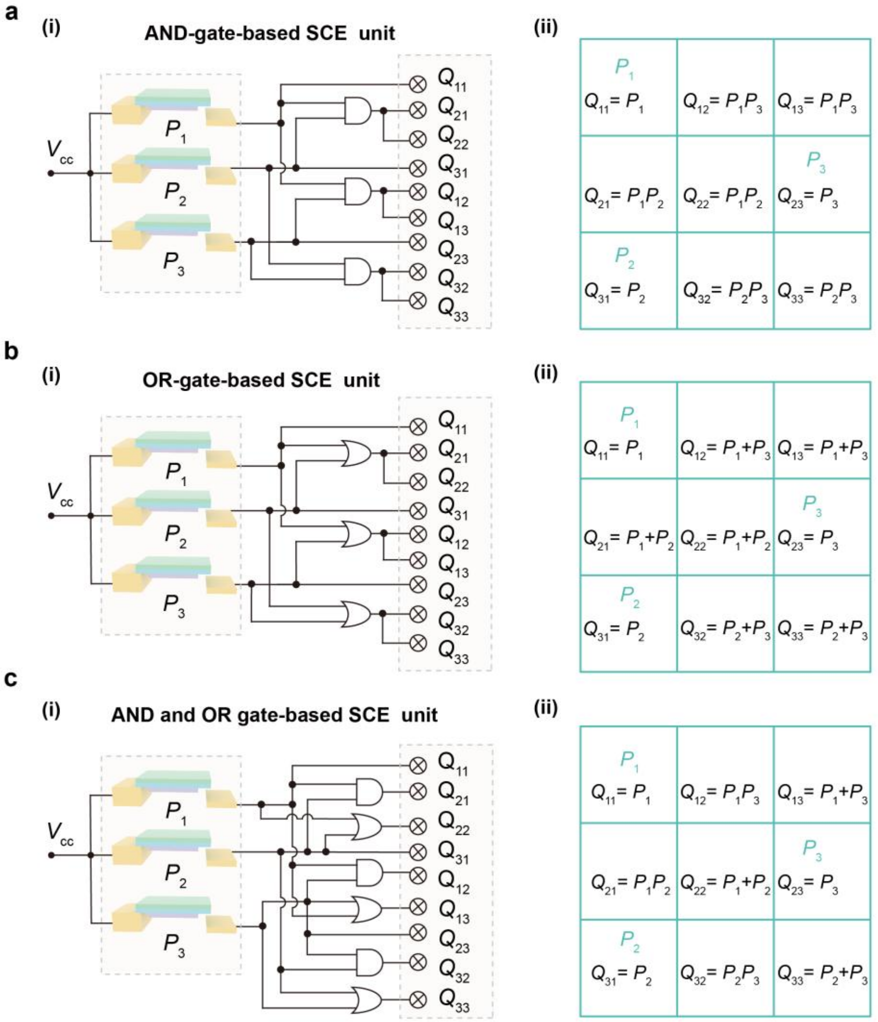

Supplementary Fig. 26. Circuit diagrams and spatial distributions of the sensing–computation–emission (SCE) unit for reconstruction schemes. a-c (i) Circuit diagrams of the SCE for reconstruction schemes based on AND logic (**a**), OR logic (**b**), and the combined AND/OR logic configuration (**c**). **(ii)** The spatial distributions of the sensed optical input signals (P_1, P_2, and P_3) and the corresponding output signals (Q_{11}, Q_{12}, …, Q_{33}) within the SCE unit. In panels **(a)** and (**b**), two pixels emit identical signals, specifically $Q_{12} = Q_{13}$, $Q_{21} = Q_{22}$, and $Q_{32} = Q_{33}$. In contrast, in panel (**c**), the signals emitted by different pixels are not identical, and all nine output signals are mutually independent.

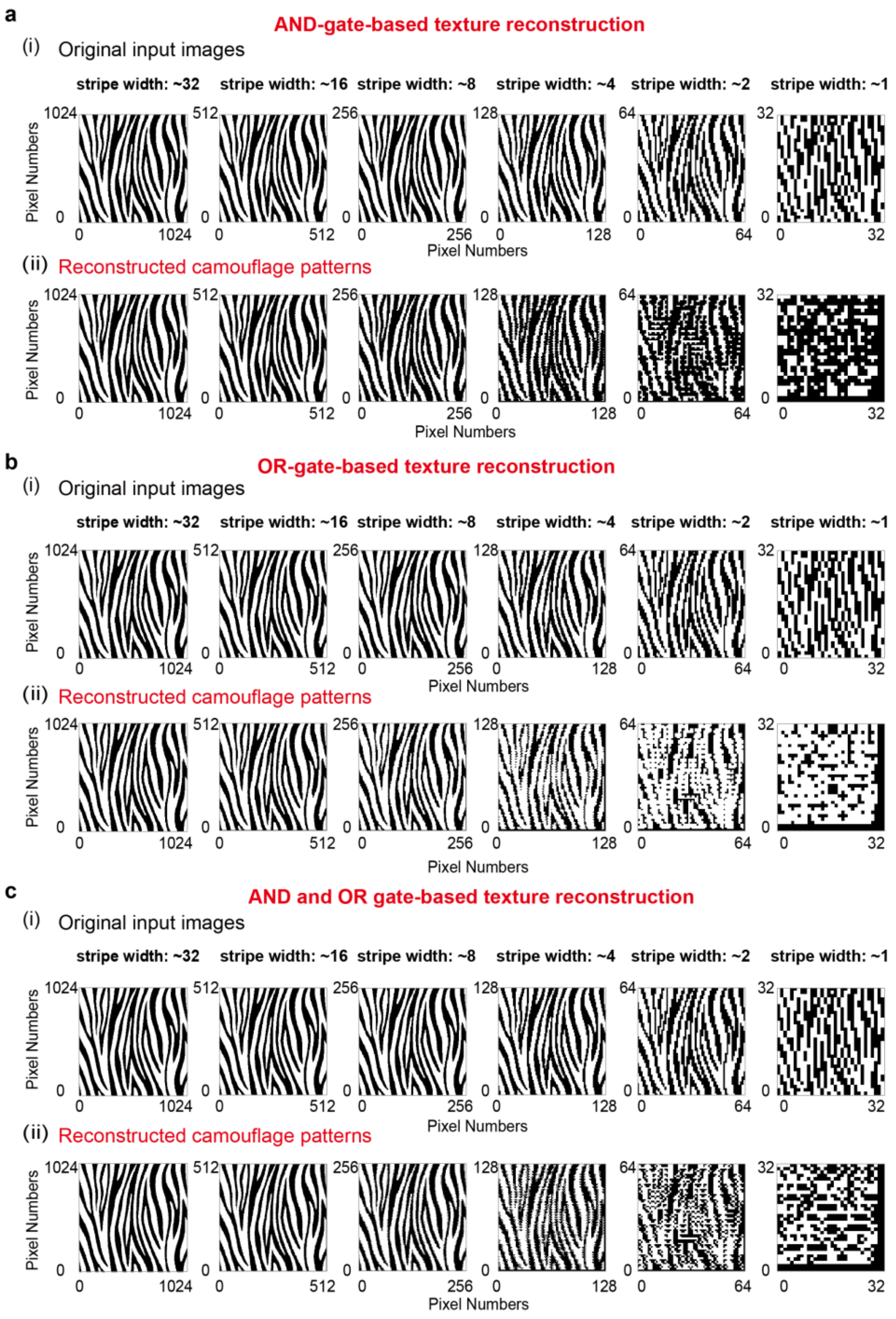

Supplementary Fig. 27. Image reconstruction of zebra textures based on different sensing–computation–emission (SCE) units. Image reconstruction using an AND-gate-based SCE unit **(a)**, an OR-gate-based SCE unit **(b)**, and a combined AND/OR gate-based SCE unit **(c)** for textures with different average zebra-stripe widths. For an input resolution of 1024 × 1024, the average stripe width corresponds to approximately 32 pixels; for 512 × 512, approximately 16 pixels; for 256 × 256, approximately 8 pixels; for 128 × 128, approximately 4 pixels; for 64 × 64, approximately 2 pixels; and for 32 × 32, approximately 1 pixel.

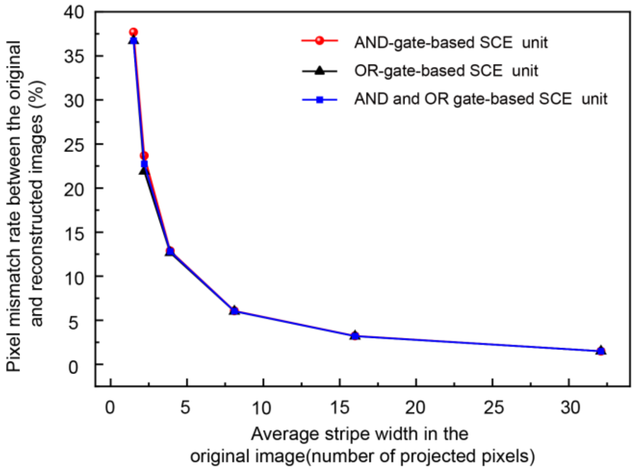

Supplementary Fig. 28. Pixel-level differences in zebra-stripe textures reconstructed using different sensing–computation–emission (SCE) units. Pixel mismatch rates between the original and reconstructed images for zebra textures with varying average stripe widths, using an AND-gate-based SCE unit (Supplementary Fig. 27a), an OR-gate-based SCE unit (Supplementary Fig. 27b), and a combined AND/OR gate-based SCE unit (Supplementary Fig. 27c). Here, pixel mismatch rate between the original and reconstructed images is defined as the ratio of pixels with inconsistent brightness to the total number of pixels.

(2) Clarify whether such redundancy is intentional, a constraint inherent to the present circuitry, or something that could be eliminated by incorporating additional logic operations (e.g., OR, NOT, or mixed logic)

The redundancy in the current logic architecture is an intentional design choice rather than an intrinsic limitation of the circuitry. Our guiding principle aims to achieve the required camouflage performance using the minimal amount of sensing and computational resources. We therefore adopted an image reconstruction scheme based on AND gates because it already provides sufficient capability to reconstruct the spatial light–dark gradient features extracted from the sensing units, without necessitating additional logic functions.

To further substantiate this point, we evaluated the reconstruction performance of alternative logic operations. We found that using OR logic alone (Supplementary Fig.26b) or in combination with AND logic (in this case, each pixel within the 3 × 3 SCE output region encodes independent information; Supplementary Fig.26c) can produce camouflage patterns which are essentially identical to those generated by AND logic in terms of representational capacity and fidelity (Supplementary Fig. 27b and

Supplementary Fig. 27c). In all cases, the performance is governed by the same fundamental factor: the relative scale between the texture-feature size and the spatial extent of the image perceived by a 3 × 3 SCE unit (Supplementary Fig.28). High-fidelity reconstruction is achieved only when the texture feature size is considerably larger than the effective spatial coverage of the sensing window, and this conclusion holds irrespective of whether an AND-gate or OR-gate logic is employed.

In contrast, logic operations involving inversion (e.g., NOT, NAND, NOR) are inappropriate because the negation operation reverses the local intensity-gradient polarity, converting bright regions to dark and vice versa, and thus introduces large perceptual errors in the reconstructed patterns.

These observations demonstrate that the use of AND logic reflects a deliberate and efficient architectural choice rather than a functional constraint. It minimizes hardware resource consumption while still offering adequate representational capacity for global texture reconstruction.

(3) Clarifying this structural issue is important for an accurate assessment of the system's adaptive camouflage capabilities.

In our architecture, the fidelity of reconstructed camouflage patterns is primarily governed by the relationship between the characteristic texture size and the spatial region perceived by an individual SCE unit. High-fidelity reconstruction is achieved when the texture features are substantially larger than the effective sensing window of the SCE unit. When the feature size approaches the spatial scale sampled by the unit, the reconstruction fidelity is necessarily affected. This behavior follows directly from the operating principle of the SCE, which is designed to resolve spatial intensity gradients that extend beyond the 3 × 3 sampling window.

The redundancy introduced by the use of pure AND logic is an intentional design choice rather than a circuit limitation. AND logic is sufficient to extract the brightness-gradient information required for camouflage reconstruction, and replacing it with OR logic or a hybrid OR/AND configuration does not yield additional representational benefits. Logic operations involving inversion (such as NOT, NAND, or NOR) are not appropriate in this context because they reverse the local contrast polarity, resulting in substantial perceptual distortion in the reconstructed patterns. Taken together, these considerations define a minimal yet fully sufficient logic scheme that is optimized for resource-efficient operation.

Simulations based on zebra-stripe textures quantitatively support this conclusion. When the stripe width exceeds approximately four pixels, the pixel mismatch rate remains below ~15%. As the feature size is further reduced to become comparable to the 3 × 3 sensing region, the mismatch rate increases, and the reconstructed images gradually lose their texture-camouflage capability.

Overall, these results show that the architecture follows a lightweight design philosophy consistent with hardware efficiency considerations, and that its operational range and limitations can be reliably assessed through the relative scale between scene texture features and the effective sensing area of the SCE unit.

Supplementary Note 8. Comparison of Material Design and Computational Approach with Previous Studies

a Comparison of Material Engineering

To emphasize the materials engineering contribution of our work, we conducted a comparative analysis between our PCF (PDMS/Ag NWs/PANi–PNIPAm) actuator and representative light-responsive actuating materials reported in the literature (Supplementary Table 2). The comparison is presented in terms of (1) responsiveness, (2) durability, and (3) energy efficiency, as detailed below.

(1) Responsiveness

Most previously reported photothermal actuators require tens of seconds to minutes to complete a single actuation event (e.g., 2 min for CdS QDs[15], ~40 s for fluorophore-grafted PNIPAm[23], and ~20 s for graphene QDs–PNIPAm[24]). Even advanced nanocomposite systems such as CNT–LCE and MXene/PEDOT:PSS achieve response times of ~11–12 s[16,25], but only under high light intensities.

In contrast, our PCF actuator exhibits a significantly accelerated response, achieving a rapid actuation time of 1.04 s under 130 mW/cm^2 and an ultrafast response of 0.13 s under 300 mW/cm^2. This represents an improvement of over two orders of magnitude compared with state-of-the-art photothermal systems, confirming the exceptional responsiveness of our materials design.

(2) Durability

Reported photothermal actuators typically demonstrate limited operational lifetimes, with stable performance over 5–500 cycles before mechanical fatigue or irreversible degradation occurs[8-12]. In comparison, our actuator maintains stable performance over 600 cycles without notable performance decay, indicating robust

mechanical durability and material reliability.

(3) Energy Efficiency

Our PCF system demonstrates a photothermal conversion efficiency (η) of 49.6%, defined as the ratio between absorbed optical energy (E_{abs}) and the energy converted to heat (E_{heat}). While previous studies did not report η values, the incident energy density (Ed_{in}) can be estimated from the light intensity and response time of each system. Earlier photothermal actuators generally require very high light intensities (up to 5 W/cm^2)[10] and large energy density inputs (>10 J cm^{-2}, occasionally exceeding 100 J cm^{-2} per switching event)[8, 10, 11]. In sharp contrast, our system requires only 39.4 mJ cm^{-2} per switching event, which is several orders of magnitude lower than most reported systems, underscoring its high energy efficiency.

In summary, these comparative analyses highlight the advantages of our materials engineering strategy, which synergistically combines PANi as a photothermal transducer, PNIPAm for rapid and reversible phase transition, and PDMS for mechanical actuation. This integrated design achieves a unique combination of ultrafast responsiveness, exceptional durability, and high energy efficiency, establishing our PCF as a promising platform material for light-driven mechanical computing and adaptive intelligent devices.

b Comparison of Computational Approaches

To further clarify the distinction between metamaterial-based mechanical computing and our material-based approach, we analyzed the following trade-offs:

(1) Switching speed and delay.

Metamaterial systems are mechanically robust but inherently slow, whereas material-based systems are considerably faster and have clear potential for further acceleration.

Metamaterial logic typically operates on the scale of seconds to minutes (for example, 2–3 s response time for rotating blocks (with no recovery time reported)[13, 14], 5–20 s response time for hydrogel gates (with no recovery time reported)[15], approximately 27 s switching time for electrically driven systems[16], and up to 80 minutes response time for thermal adders (with no recovery time reported) [17]). These delays are mainly caused by inertia and elastic recovery, which make sub-second response time particularly difficult. In contrast, our system achieves a significantly accelerated switching performance. Under 300 mW/cm^2 illumination, the response time of 0.13 s (Supplementary Fig. 6a) is over an order of magnitude faster than that of most

existing systems. This improvement stems from the light–thermal–mechanical coupling mechanism. The system's overall switching time is less than 3 s, enabling stable operation at a practical frequency of approximately 0.3–0.4 Hz, outperforming previous studies systems[9, 16, 18]. With further miniaturization, the switching time is expected to decrease further, enabling even faster logic operations.

(2) Manufacturing complexity and scalability.

Metamaterial approaches allow intuitive prototyping but face serious limitations in scaling, while material-based systems are inherently suitable for integration and large-scale architectures but demand advanced fabrication precision.

Metamaterials are usually fabricated through 3D printing or modular assembly. This approach is convenient for proof-of-concept demonstrations but generally produces bulky centimeter-scale arrays with low integration density and limited scalability. Our thin-film strategy, in contrast, is intrinsically compatible with miniaturization, multilayer stacking, and wafer-scale integration. For instance, we demonstrated a 2-bit adder, highlighting the feasibility of scalable circuit design, although precise fabrication and strict material uniformity are still required.

(3) Reconfigurability and environmental responsiveness.

Metamaterial-based systems offer convenient fabrication but limited adaptability due to their reliance on predefined structures, whereas material-based systems enable stimulus-driven reconfigurability with higher adaptability at the cost of increased fabrication complexity.

In metamaterial systems, reconfigurability is typically achieved through geometric multistability, such as folding, rotation, or compression. Most reported systems rely on external mechanical resetting, which leads to slow response and poor adaptability to multiple stimuli. In contrast, our material-based devices exploit the intrinsic sensitivity of functional materials to light, temperature, and humidity, enabling dynamic reconfiguration of information processing behaviors. The devices maintain stable operation over more than 600 switching cycles, confirming their reproducibility and durability. Nevertheless, the practical implementation of this technology relies on developing highly uniform, responsive composite films and precision mechanical fabrication techniques, which remain essential challenges for large-scale applications.

Supplementary Table 2. Comparison of selected materials with representative alternatives

Alternatives	Responsiveness	Durability	Incident energy density Ed_{in}	Energy Efficiency	References
CdS QDs	2 min (100 mW/cm^2 for 365 nm;120 mW/cm^2 for 600-1200 nm)	10 cycles	12-14.4 J/cm^2	N/A	Ref[8]
Fluorophore-grafted P(NIPAM-co-allylamine)	40 s (0.15-0.2 W/ cm^2, 405/502/638 nm)	6 cycles	6-8 J/cm^2	N/A	Ref[12]
Graphene QDs-PNIPAm	20 s (5 W/ cm^2, 808 nm)	5 cycles	100 J/cm^2	N/A	Ref[10]
CNT-LCE	12 s (370.7 mW,1545 nm)	500 cycles	4.45 J/ cm^2	N/A	Ref[9]
MXene/PEDOT:PSS-Integrated NIPAAM	11.3 s (0.9 W/cm^2 ,808 nm)	300 cycles	10.2 J/cm^2	N/A	Ref[11]
PDMS/AgNWs/PANi–PNIPAm	1.04 s (130 mW/cm^2, 650 nm);<br>0.13 s (300 mW/cm^2)	600 cycles	39.4 mJ/ cm^2	49.6%	This work

Supplementary Table 3. Quantitative Benchmark of Key Performance Metrics for Stimuli-Responsive Logic Systems

External Stimuli	Response Time	Recovery Time	Switching Time	Operating frequency	Power Consumption	Scalability	Environmental Resilience	Ref.
**Mechanical force**	Second-level (Estimated from the movie)	N/A	N/A	N/A	Zero static power	Seven fundamental logic gates: AND, OR, XOR, NOT, NAND, NOR, NXOR; logic gate combination OR-NAND, NOR-AND, OR-AND, and AND-NAND	N/A	El Helou[13]
**Mechanical force**	Second-level (Estimated from the movie)	N/A	N/A	N/A	Zero static power	Seven fundamental logic gates: AND, OR, XOR, NOT, NAND, NOR, NXOR; 2-bit Adder/Subtractor/Multiplier; 4-bit Magnitude Comparator	N/A	El Helou[14]
**Mechanical force**	Second-level (Estimated from the movie)	N/A	N/A	N/A	Zero static power	NOT, AND, OR; Supports cascaded logic (e.g., 3-level AND/OR/NOT networks)	N/A	Byun[15]
**Mechanical force**	Second-level (Estimated from the movie)	N/A	N/A	N/A	Zero static power	AND, OR, NOT; Full Adder; Cascading of multiple (7) adders	Operates at -20°C for >24 hours	Wu[19]
**Mechanical force**	N/A	N/A	N/A	N/A	Self-powered (Power generation: 4.35 nW)	Six basic logic gates (AND, OR, XOR, NAND, NOR, XNOR) full adder; 3-bit ternary computing	N/A	Zhang[20]
**Electromagnet**	Second-level	N/A	N/A	N/A	Zero static power	NOR, NAND, NOT, OR, AND; Half adder;	N/A	Mei[21]

	(Estimated from the movie)					3D printable at multiple scales (macro to micro)		
**Magnetic**	Second-level (Estimated from the movie)	N/A	N/A	N/A	Zero static power	AND, OR	Field Strength (B): -85 to 85 mT	Pal[22]
**Magnetic or mechanical**	Second-level (Estimated from the movie)	N/A	N/A	N/A	Zero static power	AND, OR, NOR, NAND; Maximum demonstrated: 30 × 27 unit array	Compression: Withstands 2.8 kPa pressure at 2% strain	Li[23]
**Solvents (toluene and water)**	0.6 to 108 s	70 min	420.6 to 528 s	0.0019 to 0.0024 Hz	Zero static power	AND, OR, NAND;	Operates in liquid environments (water/toluene) Reusable after 70min drying	Jiang[18]
**Humidity**	N/A	N/A	N/A	N/A	Zero static power	SR Latch, AND, OR; Majority Gate;	Operates in 0-85% RH range; 5 cycles	Treml[24]
**Electrical**	N/A	N/A	27.2 s	0.0368 Hz	5.5 W (1-bit system) 7.4 W (8-bit solver)	1-bit (T flip-flop); 2-bit (counter); 8-bit (equation solver)	N/A	El Helou[16]
**Thermal**	Time Constant (τ):160 minutes; 0.5 τ (full adder)	N/A	N/A	N/A	Zero static power	NOT, AND, OR, XOR; SR Latch; Full adder	continuous operation over 1000 min; Vacuum-compatible	Chen[17]
**Vibration**	N/A	N/A	N/A	N/A	Zero static power	AND, OR, XOR, NOT, NAND, NOR, NXOR; Half Adder;	Signal Leakage (OFF state): < 10% of max signal;	R. Bilal[25]

						Full Adder	Signal Clarity (ON/OFF difference): > 10	
**Light**	2 min (120 mW/cm^2)	N/A	N/A	N/A	Zero static power	AND, NOT;	100 mW to 397.7 mW	Peng[8]
**Light**	12 s (379.7 mW)	15 s	27 s	0.037 Hz	Zero static power	AND, OR, NOT;<br>Soft optical switch array	500 cycles	Wang[9]
**Light**	~1.04 s (130 mW/cm^2)<br>~0.13 s (300 mW/cm^2)	~1.93 s (130 mW/cm^2)<br>~2.4 s (300 mW/cm^2)	~2.97 s (130 mW/cm^2)<br>~2.53 s (300 mW/cm^2)	~ 0.3 Hz (130 mW/cm^2)<br>~ 0.4 Hz (300 mW/cm^2)	Zero static power	AND, OR, NOT, XOR;<br>2-bit Adder;	Light intensity: 70–310 mW/cm^2;<br>Humidity: 47–86% RH<br>Temperature: 10–30 °C;<br>Cycles: 600	This Work

(The data for our work in the table are derived from the averaged values shown in Supplementary Fig. 6a.)

Supplementary References

1. Yan X, Chen Q, Huo Z, Zhang N, Ma M. Programmable Multistimuli-Responsive and Multimodal Polymer Actuator Based on a Designed Energy Transduction Network. *ACS Appl. Mater. Interfaces* **14**, 13768–13777 (2022).
2. Das A, Babu A, Chakraborty S, Van Guyse JFR, Hoogenboom R, Maji S. Poly(N-isopropylacrylamide) and Its Copolymers: A Review on Recent Advances in the Areas of Sensing and Biosensing. *Adv. Funct. Mater.* **34**, 2402432 (2024).
3. Tian K*, et al.* Stretchable Multifunctional Polydimethylsiloxane Composites with Cage-Like Conductive Architecture for Integrated Thermosensitive and Electromagnetic Interference Shielding Performance. *Adv. Funct. Mater.* **34**, 2400288 (2024).
4. Ogieglo W*, et al.* n-Hexane induced swelling of thin PDMS films under non-equilibrium nanofiltration permeation conditions, resolved by spectroscopic ellipsometry. *J. Membr. Sci.* **437**, 313–323 (2013).
5. Toshikazu TAKIGAWA TY, KatsunOri TAKAHASHI,and Toshiro MASUDA. Deswelling Kinetics of Poly(N-isopropylacrylamide) Gels atVolume-Phase Transition. *Polym. J.* **31**, 595–598 (1999).
6. Yang C*, et al.* Silver Nanowires: From Scalable Synthesis to Recyclable Foldable Electronics. *Adv. Mater.* **23**, 3052–3056 (2011).
7. Zhu D, Huang G, Zhang L, He Y, Xie H, Yu W. Silver Nanowires Contained Nanofluids with Enhanced Optical Absorption and Thermal Transportation Properties. *Energy Environ. Mater.* **2**, 22–29 (2019).
8. Peng X, Li H, Xu J, Lan C, Liu J, Wu B. Reprogrammable shape morphing hydrogel modulated by synergistic photochromism and photoactuation. *Chem. Eng. J.* **511**, 162103 (2025).
9. Wang C, Wu H, Niu Q, Yan X, Wang X. Light-Controlled Soft Switches for Optical Logic Gate Operations. *Sensors* **25**, 2051 (2025).
10. Wang Y, Zhang Z, Chen H, Zhang H, Zhang H, Zhao Y. Bio-inspired shape-memory structural color hydrogel film. *Sci. Bull.* **67**, 512–519 (2022).
11. Xue P*, et al.* Highly Conductive MXene/PEDOT:PSS-Integrated Poly(N-Isopropylacrylamide) Hydrogels for Bioinspired Somatosensory Soft Actuators. *Adv. Funct. Mater.* **33**, 2214867 (2023).
12. Koo HB, Yeon H, Bin Yoon Y, Lee TJ, Chang YT, Chang JB. Rewritable wavelength-selective hydrogel actuators grafted with fluorophores. *Mater. Horiz.* **12**, 2255–2266 (2025).
13. El Helou C, Buskohl PR, Tabor CE, Harne RL. Digital logic gates in soft, conductive mechanical metamaterials. *Nat. Commun.* **12**, 1633 (2021).
14. El Helou C, Grossmann B, Tabor CE, Buskohl PR, Harne RL. Mechanical integrated circuit materials. *Nature* **608**, 699–703 (2022).
15. Byun J, Pal A, Ko J, Sitti M. Integrated mechanical computing for autonomous soft machines. *Nat. Commun.* **15**, 2933 (2024).
16. El Helou C, Hyatt LP, Buskohl PR, Harne RL. Intelligent electroactive material systems with self-adaptive mechanical memory and sequential logic. *Proc. Natl. Acad. Sci.* **121**,

e2317340121 (2024).

17. Chen H*, et al.* Thermal Computing with Mechanical Transistors. *Adv. Funct. Mater.* **34**, 2401244 (2024).

18. Jiang Y, Korpas LM, Raney JR. Bifurcation-based embodied logic and autonomous actuation. *Nat. Commun.* **10**, 128 (2019).

19. Wu L*, et al.* Mechanical Metamaterials for Handwritten Digits Recognition. *Adv. Sci.* **11**, e2308137 (2024).

20. Zhang Q*, et al.* Meta-mechanotronics for self-powered computation. *Mater. Today* **65**, 78–89 (2023).

21. Mei T, Meng Z, Zhao K, Chen CQ. A mechanical metamaterial with reprogrammable logical functions. *Nat. Commun.* **12**, 7234 (2021).

22. Pal A, Sitti M. Programmable mechanical devices through magnetically tunable bistable elements. *Proc. Natl. Acad. Sci.* **120**, e2212489120 (2023).

23. Yanbin Li SY, Haitao Qing, Yaoye Hong, Yao Zhao, Fangjie Qi, Hao Su*, Jie Yin*. Reprogrammable and reconfigurable mechanical computing metastructures with stable and high-density memory. *Sci. Adv.* **10**, eado6476 (2024).

24. Treml B, Gillman A, Buskohl P, Vaia R. Origami mechanologic. *Proc. Natl. Acad. Sci.* **115**, 6916–6921 (2018).

25. Bilal OR, Foehr A, Daraio C. Bistable metamaterial for switching and cascading elastic vibrations. *Proc. Natl. Acad. Sci.* **114**, 4603–4606 (2017).
