# Supplementary material for: Light-programmable mechanical computing via polyaniline composite film
Source: Nat Commun. 2026 Mar 16;17:4011. doi: 10.1038/s41467-026-70425-z (PMC13136495; doi:10.1038/s41467-026-70425-z)
Supplement: Supplementary file 2 — Description of Additional Supplementary Files [file 41467_2026_70425_MOESM2_ESM.pdf]

Description of Additional Supplementary Files

File name: **Supplementary Movie 1**

Description: Light-programmable mechanical deformation of the PCF

File name: **Supplementary Movie 2**

Description: Light-programmable mechanical deformation of the PCF and humidity-assisted recovery

File name: **Supplementary Movie 3**

Description: Logical operation of the SPST relay and its reset by humidity

File name: **Supplementary Movie 4**

Description: Electrical bonding between AND gate and PI flexible circuit board using SEBS connections

File name: **Supplementary Movie 5**

Description: Light-programmable AND logic operation

File name: **Supplementary Movie 6**

Description: Light-programmable XOR logic operation

File name: **Supplementary Movie 7**

Description: Full-adder construction via self-adhesive SEBS connections

File name: **Supplementary Movie 8**

Description: Light-programmable full adder operation

File name: **Supplementary Movie 9**

Description: Humidity control system

File name: **Supplementary Movie 10**

Description: Adaptive texture camouflage demonstrated using a 3 × 3 SCE unit

File name: **Supplementary Code**

Description: MATLAB scripts were used to simulate the adaptive camouflage functionality presented in Fig. 4 of the main text, to evaluate system robustness under damage scenarios (Supplementary Fig. 25), and to validate its texture resolution capability (Supplementary Fig. 27 and Fig. 28).
