# Supplementary figures and images for: Light-programmable mechanical computing via polyaniline composite film
Source: Nat Commun. 2026 Mar 16;17:4011. doi: 10.1038/s41467-026-70425-z (PMC13136495; doi:10.1038/s41467-026-70425-z)

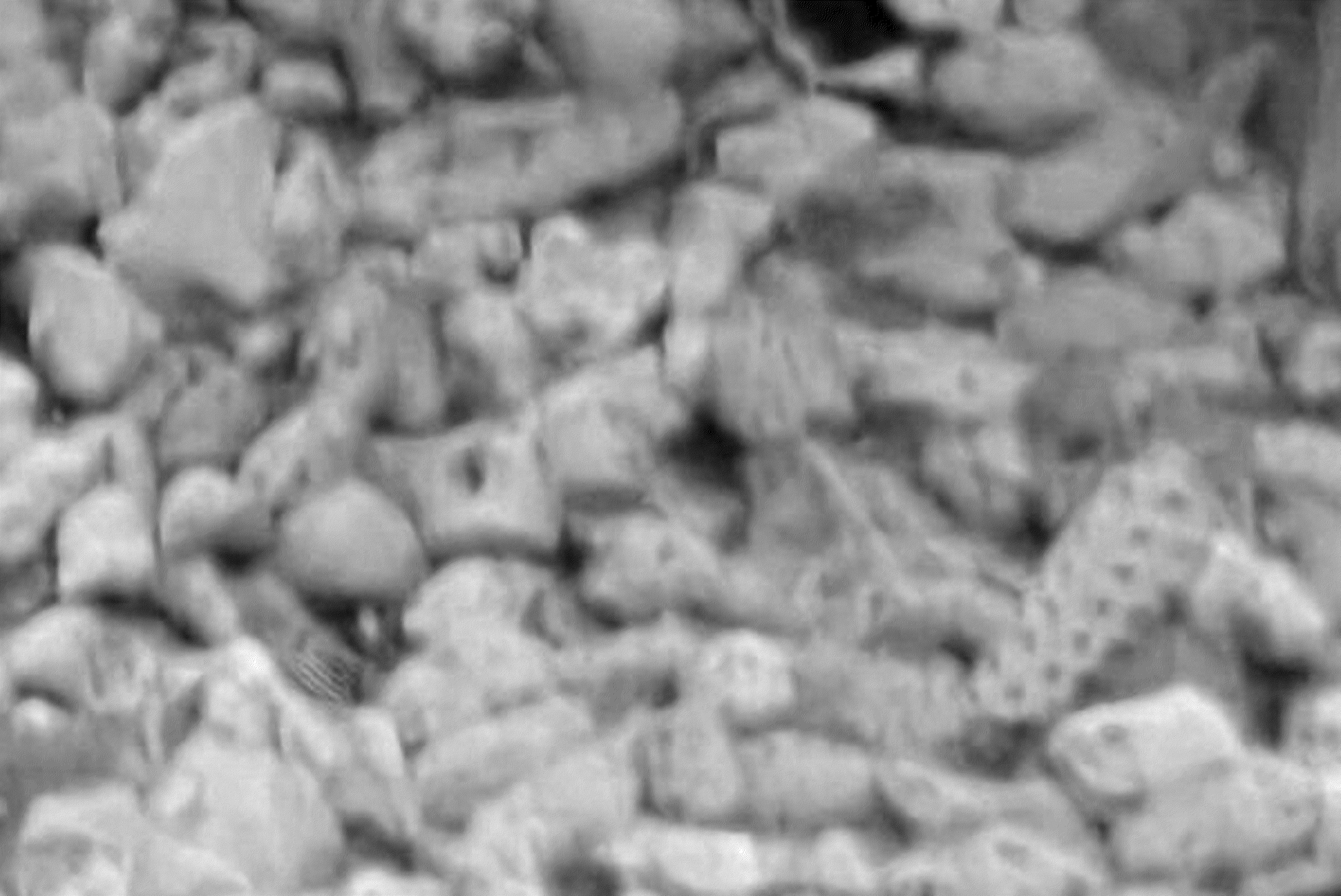

Supplement: Supplementary file 13 — Supplementary Code [file 41467_2026_70425_MOESM13_ESM.zip › Supplementary Code/Dither_PC_10.jpg]

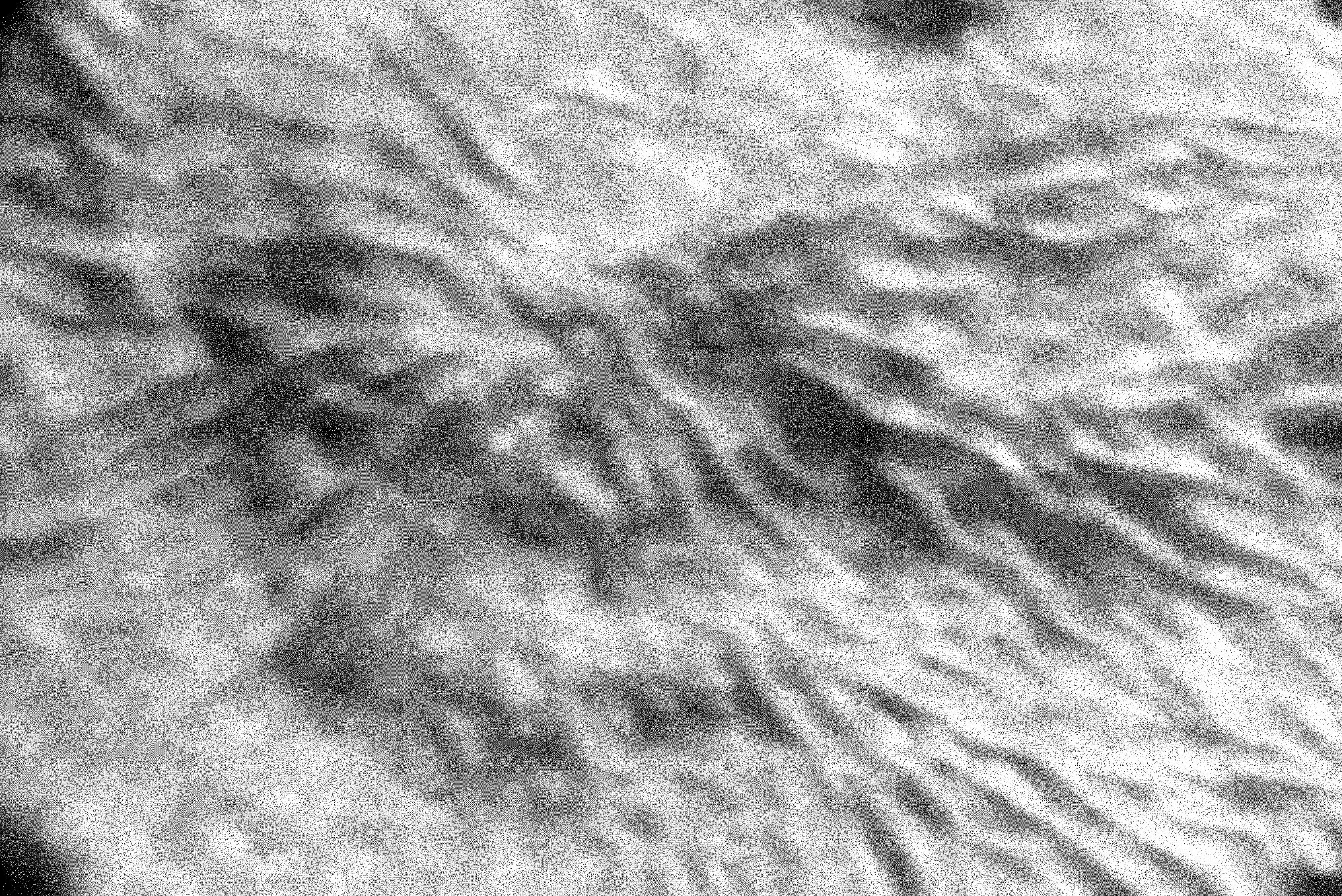

Supplement: Supplementary file 13 — Supplementary Code [file 41467_2026_70425_MOESM13_ESM.zip › Supplementary Code/Dither_PC_11.jpg]

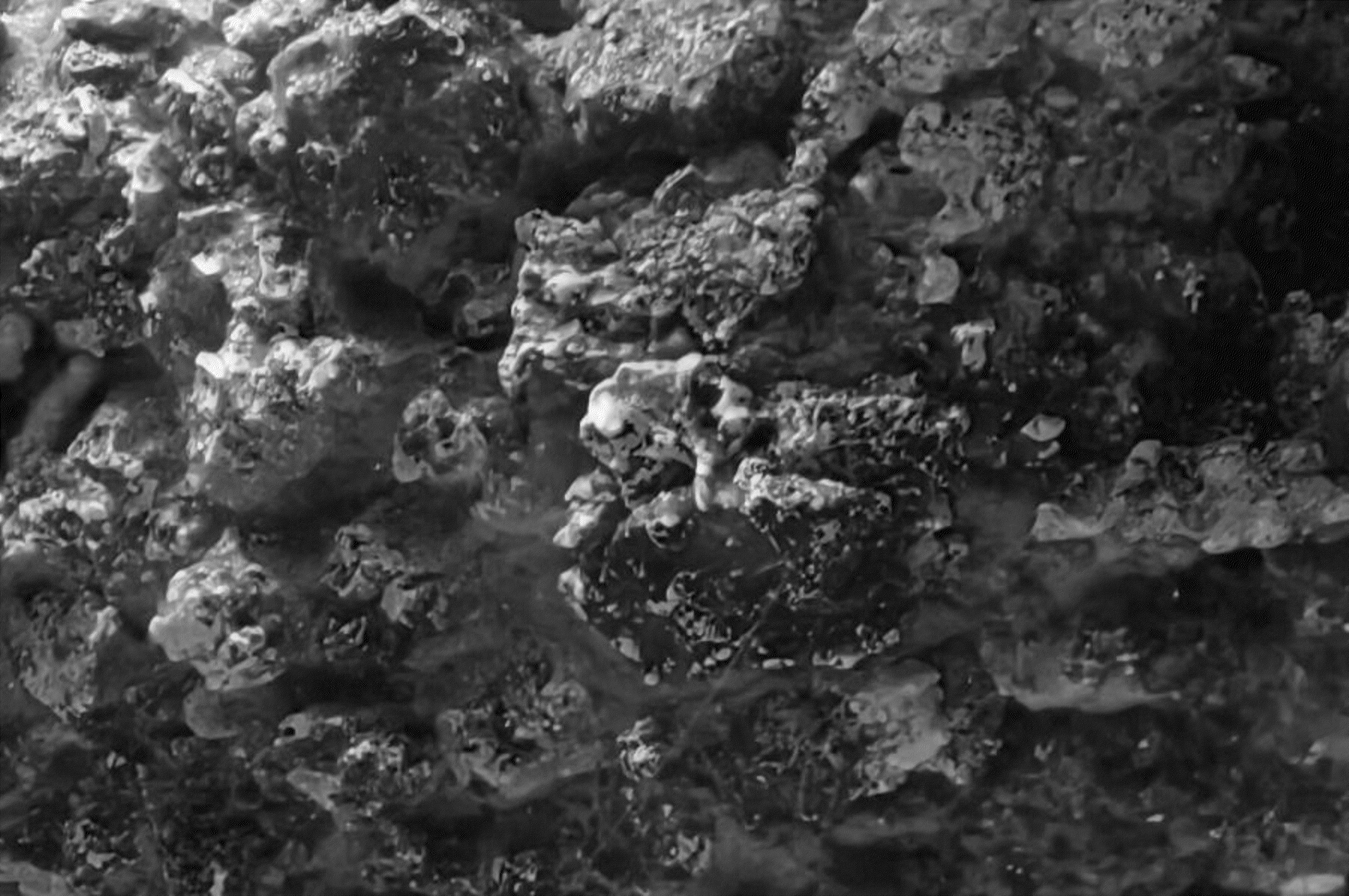

Supplement: Supplementary file 13 — Supplementary Code [file 41467_2026_70425_MOESM13_ESM.zip › Supplementary Code/Dither_PC_12.jpg]

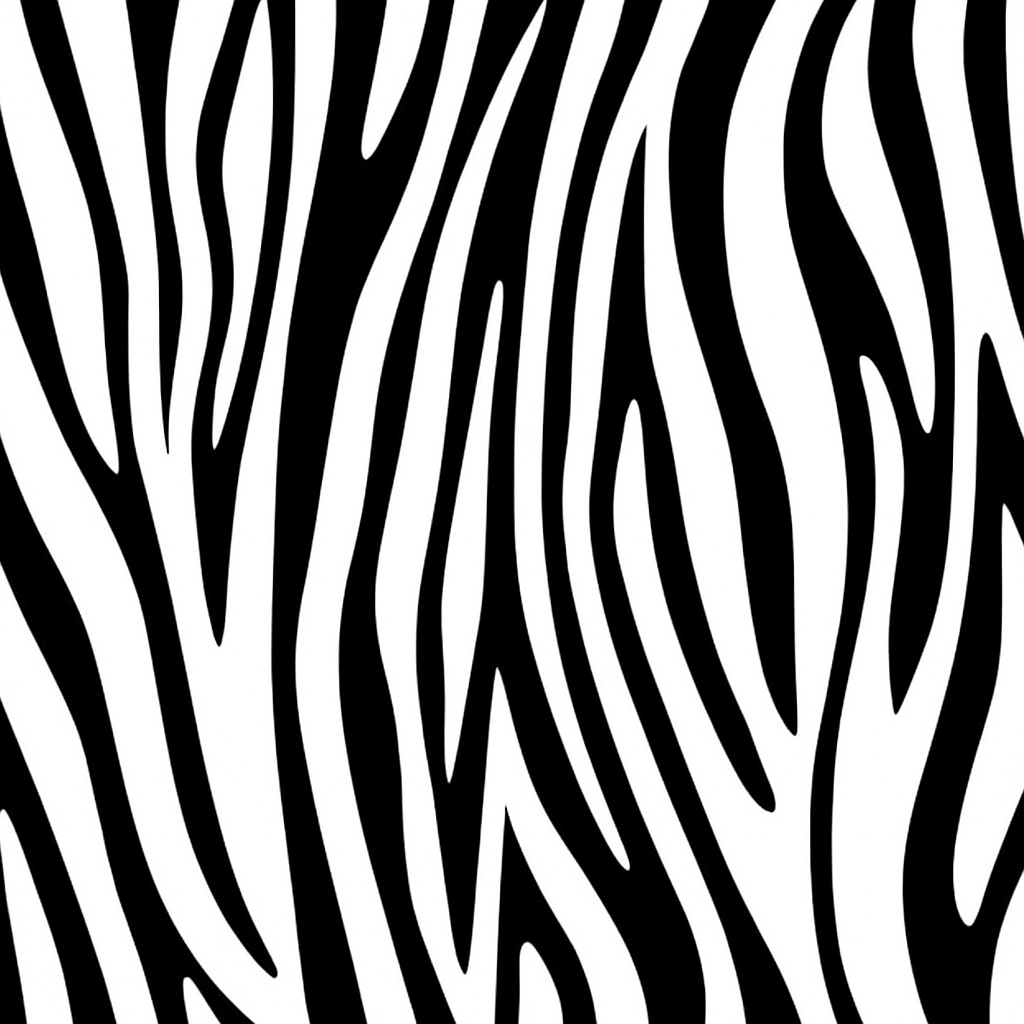

Supplement: Supplementary file 13 — Supplementary Code [file 41467_2026_70425_MOESM13_ESM.zip › Supplementary Code/zebra-3.png]
